# Mapping interactions of calmodulin and neuronal NO synthase by crosslinking and mass spectrometry

**DOI:** 10.1016/j.jbc.2023.105464

**Published:** 2023-11-16

**Authors:** Dana Felker, Kanghyun Lee, Thomas H. Pospiech, Yoshihiro Morishima, Haoming Zhang, Miranda Lau, Daniel R. Southworth, Yoichi Osawa

**Affiliations:** 1 Department of Pharmacology, University of Michigan Medical School, Ann Arbor, Michigan, USA; 2 Institute for Neurodegenerative Diseases, University of California, San Francisco, San Francisco, California, USA; 3 Department of Biochemistry and Biophysics, University of California, San Francisco, San Francisco, California, USA

**Keywords:** nitric oxide synthase, crosslinking, calmodulin, mass spectrometry, protein-protein interactions

## Abstract

Neuronal nitric oxide synthase (nNOS) is a homodimeric cytochrome P450-like enzyme that catalyzes the conversion of L-arginine to nitric oxide in the presence of NADPH and molecular oxygen. The binding of calmodulin (CaM) to a linker region between the FAD/FMN-containing reductase domain, and the heme-containing oxygenase domain is needed for electron transfer reactions, reduction of the heme, and NO synthesis. Due to the dynamic nature of the reductase domain and low resolution of available full-length structures, the exact conformation of the CaM-bound active complex during heme reduction is still unresolved. Interestingly, hydrogen-deuterium exchange and mass spectrometry studies revealed interactions of the FMN domain and CaM with the oxygenase domain for iNOS, but not nNOS. This finding prompted us to utilize covalent crosslinking and mass spectrometry to clarify interactions of CaM with nNOS. Specifically, MS-cleavable bifunctional crosslinker disuccinimidyl dibutyric urea was used to identify thirteen unique crosslinks between CaM and nNOS as well as 61 crosslinks within the nNOS. The crosslinks provided evidence for CaM interaction with the oxygenase and reductase domain residues as well as interactions of the FMN domain with the oxygenase dimer. Cryo-EM studies, which gave a high-resolution model of the oxygenase domain, along with crosslink-guided docking provided a model of nNOS that brings the FMN within 15 Å of the heme in support for a more compact conformation than previously observed. These studies also point to the utility of covalent crosslinking and mass spectrometry in capturing transient dynamic conformations that may not be captured by hydrogen-deuterium exchange and mass spectrometry experiments.

Nitric oxide (NO) is well established as a cellular signaling molecule that plays a critical role in a variety of physiologic functions as well as pathological conditions. NO is a freely diffusible gaseous molecule that is produced when needed by NO synthases (NOS) *via* the metabolic conversion of L-arginine to L-citrulline in the presence of NADPH and molecular oxygen (1, 2). There are three major isoforms of NOS, one inducible isoform (iNOS) and two constitutive isoforms: one initially found in neuronal tissue (nNOS), and another initially found in vascular endothelial tissue (eNOS) (3). All isoforms comprise a heme-binding oxygenase domain, which contains the substrate binding site as well as the bound tetrahydrobiopterin (BH_4_) cofactor; and a reductase domain, which comprises the NADPH binding site as well as the FMN and FAD moieties (4).

The synthesis of NO only occurs when the NOS is a homodimer. CaM binding allows for the efficient transfer of electrons from the reductase domain of one monomer to the heme in the oxygenase domain of the neighboring monomer so that NO synthesis can occur (5). Although the exact mechanism of how these electron transfer reactions occur is still unknown, the single-particle EM derived structures of full-length nNOS, iNOS, and eNOS have enhanced our knowledge of possible dynamic conformational changes that might facilitate these reactions (6, 7, 8). For iNOS, HDX-MS studies (9) along with the EM studies (7) support the notion that both the FMN domain and the CaM interact closely with the oxygenase domain.

In stark contrast, nNOS HDX-MS studies failed to show similar interactions between the FMN domain or CaM with the oxygenase domain (10) although EM studies on nNOS indicate that similar dynamic movements of the reductase domain likely occur (6, 7). As indicated by Hanson *et al.* (10), it is possible that the highly transient nature of the interactions for nNOS relative to iNOS prevented their detection by HDX-MS studies. Thus, in the current study, to better understand these highly transient protein surface interactions on nNOS, we utilize a bifunctional crosslinking reagent disuccinimidyl dibutyric urea (DSBU) and high-resolution mass spectrometry (CXL-MS) technique to map protein-protein interactions in the CaM-bound nNOS homodimer. Seventy-four crosslinks were mapped to a published EM-derived structural model of CaM-bound full-length nNOS (6). The analysis of these results supports the existence of an nNOS conformation where the oxygenase domain is in closer proximity with the FMN and CaM, similar to that found for iNOS. Thus, we show that CXL-MS can capture a transient conformation of an enzyme that could not be detected by HDX-MS studies and highlights the utility of the crosslinking technique in providing structural information on unstable protein conformational states. Cryo-EM studies on the crosslinked and un-crosslinked CaM-bound nNOS gave high resolution 3D models of the oxygenase domain but failed to show the crosslinked residues. We utilize the latest cryo-EM and crosslinking data to generate a possible conformation of the nNOS where the heme domain is closer to the FMN domain.

## Results

### Crosslinking of the active CaM-bound nNOS homodimer with DSBU

**Figure 1:**
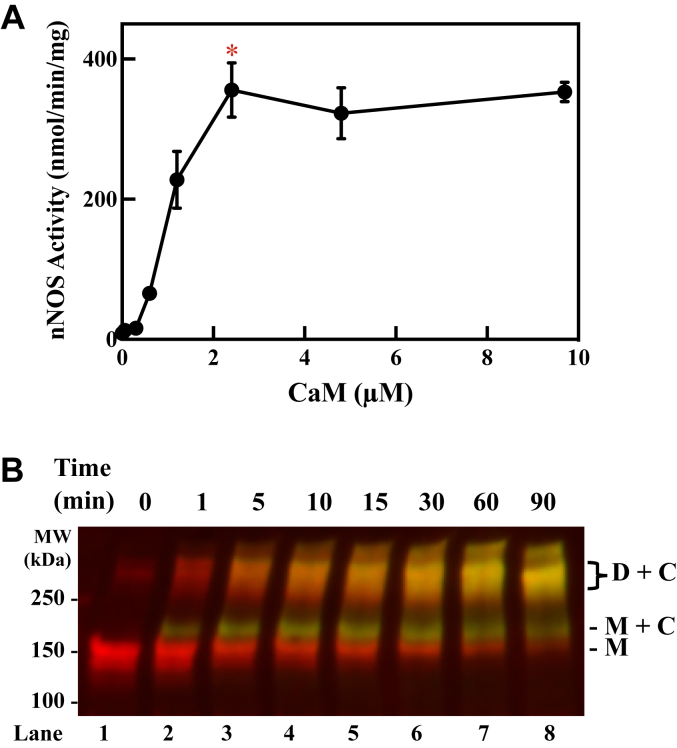
**Crosslinking of CaM to nNOS by DSBU.***A*, concentration-dependence of CaM in activating nNOS. The nNOS (2 μM) was incubated for 5 min at 4 °C with increasing concentrations of CaM in the presence of CaCl_2_ (10 μM) and BH_4_ (10 μM). Aliquots (1.28 μg) of the reaction mixtures were removed, and nNOS activity was measured as described in Experimental procedures. *Red asterisk* indicates the condition selected for subsequent crosslinking. *B*, Western blotting of DSBU-crosslinked CaM and nNOS. The nNOS (2 μM) was preincubated for 5 min at 4 °C with CaM (2.4 μM) in the presence of CaCl_2_ (10 μM) and BH_4_ (10 μM) before treatment with 0.1 mM DSBU for the designated duration. Aliquots (3 μg) of the reaction mixtures were submitted to SDS-PAGE, and nNOS (*red*) and CaM (*green*) were visualized by Western blot as described in Experimental procedures. Yellow bands contain both nNOS and CaM. *C*, CaM; CaM, calmodulin; *D*, dimer of nNOS; *M*, monomer of nNOS; nNOS, neuronal nitric oxide synthase.

The nNOS and CaM were expressed in *E. coli* and purified by standard chromatographic procedures as described in Methods to a purity of 92% and 93%, respectively. As shown in Figure 1*A*, CaM activated nNOS (2.0 μM) in a concentration-dependent manner. Maximal activation was achieved at a concentration of 2.4 μM, indicating the minimal amount of CaM needed to form the active tetrameric complex and a condition that would minimize the amount of excess free CaM for our subsequent crosslinking experiments. Next, this active CaM-bound nNOS homodimer was crosslinked with 0.1 mM DSBU and the reaction was quenched at different durations of time and analyzed by Western blotting. DSBU is a symmetric amine-reactive cross-linker designed to crosslink lysine residues but is also known to crosslink Ser, Thr, and Tyr residues if they are within approximately 27 Å (11, 12, 13). We simultaneously probed for nNOS (*red*) and CaM (*green*) to look for yellow bands indicating an overlap of these signals (Fig. 1*B*). As the native CaM-bound nNOS dimer is non-covalently associated, analysis of the reaction mixture initially shows the nNOS (*red*) migrating as a monomer (*lane 1, M*) with no associated CaM signal. Further durations of treatment with DSBU (*lanes 2–8*) causes the time-dependent formation of two yellow higher molecular weight bands, a minor band near 175 kDa (*M + C*) migrating directly above the nNOS monomer band (M) and a broad band migrating near the mass of the dimer of nNOS (*D + C*). The presence of CaM in these higher molecular mass bands is consistent with the initial crosslinking of CaM to nNOS monomer to form the minor band (*M + C*) as well as further crosslinking to form the nNOS dimer covalently linked to CaM (*D + C*). Due to the resolution of the gel at these high mass ranges, we do not know how many CaM molecules are bound. However, as will be shown later, the CaM signals are not present at these high molecular mass ranges when nNOS is omitted from the reaction, indicating that these yellow bands represent CaM covalently bound to nNOS.

### Time- and concentration-dependent crosslinking of CaM-bound nNOS homodimer examined by Coomassie staining

**Figure 2:**
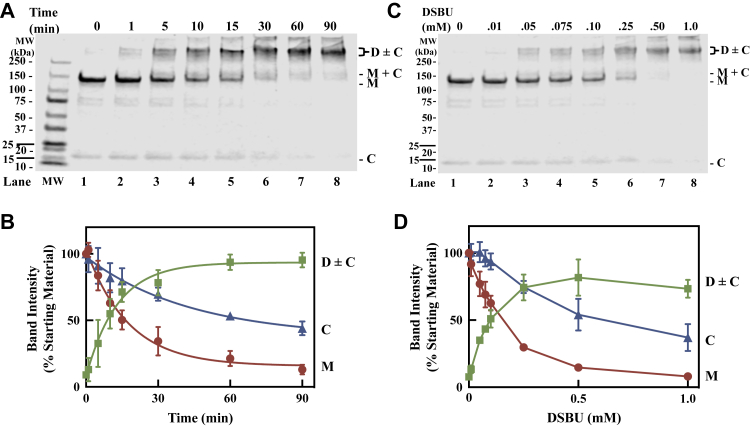
**Time- and concentration-dependent crosslinking of CaM-bound nNOS homodimer by DSBU.***A*, time-dependent formation of crosslinked nNOS and CaM after treatment with DSBU. The nNOS (2 μM) was preincubated for 5 min at 4 °C with CaM (2.4 μM) in the presence of CaCl_2_ (10 μM) and BH_4_ (10 μM) and then treated with 0.1 mM DSBU for the indicated duration. Aliquots (6 μg) of the reaction mixtures were submitted to SDS-PAGE and stained with Coomassie Blue as described in Experimental procedures. *B*, quantification of bands seen in *A*. Bands corresponding to the crosslinked dimer (*green squares*) and monomer (*red circles*), and CaM (*blue triangles*) were quantified by densitometric analysis using ImageStudio software (version 5.2) with background correction. Mean ± SD derived from three independent reaction mixtures (n = 3). The values were fit to a single exponential function in *Panel B* with the use of Prism 9 (GraphPad software, LLC). The calculated half-lives for disappearance of the monomer band and the CaM band were 12.7 ± 3.7 min and 40.6 ± 24.4 min, respectively, and were not statistically different *p* > 0.2. The appearance of the crosslinked nNOS dimer was 9.1 ± 2.5 min and was not statistically different to loss of the monomer band, *p* > 0.09. Results were analyzed using unpaired Student *t* test for comparison between two groups and considered significant at *p* < 0.05. *C*, formation of the crosslinked nNOS dimer is dependent on the concentration of DSBU. The nNOS (2 μM) was treated with the indicated concentrations of DSBU for 10 min and analyzed as in *A*. *D*, quantification of bands seen in *C*. Bands corresponding to the crosslinked dimer (*green squares*) and monomer (*red circles*), and CaM (*blue triangles*) were quantified by densitometric analysis. Background was subtracted. Mean ± SD, n = 3. Densities determined for all bands are within the linear range of detection. *C*, CaM; CaM, calmodulin; *D*, dimer of nNOS; *M*, monomer of nNOS; nNOS, neuronal nitric oxide synthase.

The blotting studies above show evidence for crosslinking of CaM to nNOS, so we examined the kinetics of the reaction in more detail to optimize conditions for MS analysis. Instead of blotting we next chose to re-examine the time course samples by SDS-PAGE and Coomassie staining as an attempt to simultaneously visualize and quantify nNOS and free CaM. As seen, the initial sample shows a major nNOS band migrating at the monomeric mass (Fig. 2*A* *lane 1, M*) as expected, in addition to a faint band around 17 kDa, which corresponds to CaM (*C*). Further treatment with 0.1 mM DSBU causes the time-dependent loss of the nNOS monomer band (*M*) and the formation of a higher mass band that likely corresponds to a mixture of crosslinked nNOS dimer and crosslinked nNOS dimer covalently linked to one or two CaM molecules (*D ± C*). The Coomassie staining for nNOS is much more prominent than CaM, so we are essentially visualizing nNOS in these bands as the crosslinking proceeds. There is a very faint band above the nNOS monomer band at 175 kDa, consistent with CaM crosslinked to nNOS (*M + C*) observed above. As shown in Figure 2*B*, we quantified the major bands corresponding to CaM (*C, blue triangle*), nNOS monomer (*M, red circle*) and the broad band containing a mixture of nNOS dimer and nNOS dimer with CaM (*D ± C, green square*). The faint band at 175 kDa was not quantified. There is a time-dependent loss of the nNOS monomer leading to the concomitant appearance of the crosslinked nNOS dimer. Consistent with this interpretation, when modeled to a single exponential function the half-life of the loss of the nNOS monomer (12.7 ± 3.7 min) was not different from that found for the appearance of the crosslinked nNOS dimer (9.1 ± 2.5 min) with *p* value > 0.09. Due to the large error in quantification of CaM, we found no statistical difference in the rate of disappearance of the monomer band when compared to the loss of the CaM band (40.6 ± 24.4 min, *p* > 0.2). As shown in Figure 2*C* (*Lanes 1–8*), the formation of crosslinked dimer and loss of CaM after 10 min of reaction is also dependent on the concentration of DSBU. As shown in Figure 2*D*, the quantitation of the major bands shows that loss of monomer is concomitant to the appearance of the dimer with the major changes occurring below 0.25 mM of DSBU. Thus, taken together, the time and concentration studies clearly show that shorter time periods and lower concentrations of DSBU are warranted for examining the initial crosslinking reaction.

### Time- and concentration-dependent crosslinking of CaM to nNOS

**Figure 3:**
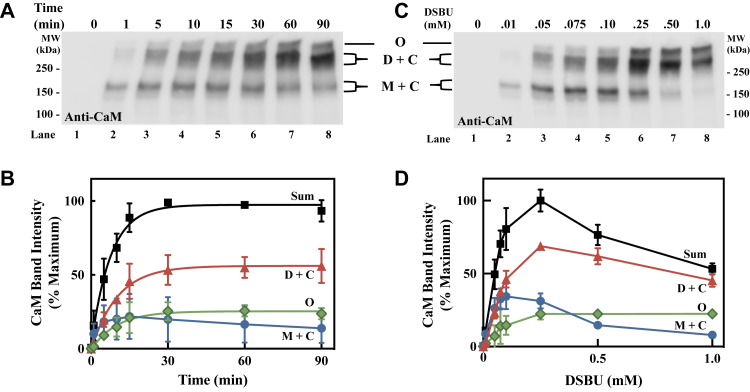
**Time- and concentration-dependent crosslinking of CaM to nNOS by DSBU.** The nNOS was treated with DSBU as in Figure 2. Aliquots (3 μg) of the reaction mixtures were analyzed for nNOS and CaM by Western blot as described in Experimental procedures. *A*, time-dependent formation of high molecular mass CaM-containing bands. Bands containing CaM crosslinked to nNOS dimer (D + C), nNOS monomer (M + C), as well as a very high molecular mass species (O) are indicated. The same blot in Figure 1*B* was reused, but in this case, only the CaM signal is shown. *B*, the CaM-containing bands in (*A*) were quantified. Bands corresponding to CaM crosslinked to the nNOS dimer (*red triangles*), nNOS monomer (*blue circles*), and higher molecular mass species (*green diamonds*) were quantified by densitometric analysis as in Figure 2*B*. Sum is the addition of all the CaM-containing bands (*black squares*). Mean ± SD, n = 3. The appearance of the CaM-bound dimer band was fit as in Figure 2*A*, and the calculated half-life is 7.1 ± 1.9 min. *C*, dependence on the concentration of DSBU in the formation of high molecular mass CaM-containing bands. Samples were prepared as in (*A*), except that the concentrations of DSBU is indicated and treatment was for 10 min. *D*, the CaM-containing bands in (C) were quantified as indicated in (*B*). Mean ± SD (n = 3). CaM, calmodulin; nNOS, neuronal nitric oxide synthase.

To determine the kinetics of CaM crosslinking more specifically to nNOS, we examined CaM by Western blotting. Due to the vastly differing transfer rates, it was not possible to look at free CaM in these blots, but we chose to carefully monitor the mass ranges corresponding to the major nNOS bands. As shown in Figure 3*A*, treatment with DSBU results in the time-dependent crosslinking of CaM to nNOS with the appearance of two major bands, an nNOS monomer linked to CaM (*M + C*) and an nNOS dimer linked to CaM (*D + C*). There was also some CaM that traveled much above the dimer of nNOS close to the stacking gel indicating high molecular mass multimers or oligomers (*O*). As shown in Figure 3*B*, the quantitation of the bands shows that formation of the CaM bound nNOS dimer *(D + C*) occurs with a half-life of 7.1 ± 1.9 min at a rate similar to that found for the formation of the crosslinked dimer above. As shown in Figure 3*C*, when examined at a 10-min reaction time, a similar pattern of CaM crosslinking to nNOS monomer and dimer is observed at lower concentrations of DSBU (*lanes, 1–5*) whereas higher concentrations give a loss of total CaM, suggesting formation of over-crosslinked products that form aggregates that no longer run on the gel. This is clearly seen when the bands are quantified (Fig. 3*D*). Thus, we found that treatment of nNOS prebound with CaM (2.4 μM) with 0.1 mM DSBU for 10 min avoided over crosslinking, aggregation, and loss of protein. Thus, this was chosen as the optimal conditions for all further studies.

### The CaM-containing crosslinked products are dependent on nNOS and calcium

**Figure 4:**
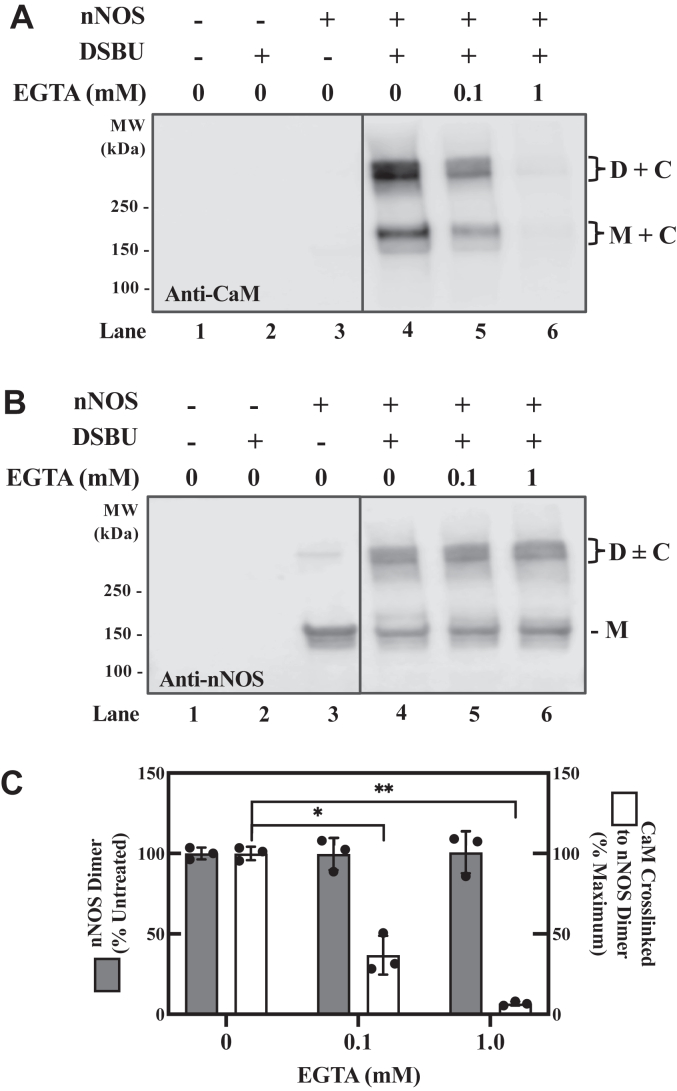
**Formation of crosslinks between CaM and nNOS is Ca/CaM-dependent.** The nNOS was treated with DSBU as described in Figure 3, except that EGTA was included in some samples as indicated. *A*, Western blot for CaM. *B*, Western blot for nNOS. *C*, EGTA abolishes CaM found in the higher molecular mass bands. Bands in lanes 4 to 6 were quantified for nNOS dimer (*solid* bars) and CaM (*open* bars). Mean ± SD, n = 3. Statistical differences were determined by unpaired *t* test. ∗*p* = 0.001; ∗∗*p* < 0.0001. The blots in A and B were spliced as indicated by *lines*. C, CaM; CaM, calmodulin; D, dimer of nNOS; M, monomer of nNOS; nNOS, neuronal nitric oxide synthase.

To ensure that the higher molecular mass crosslinked species of CaM are due to specific interactions in the CaM-bound nNOS complex, crosslinking was carried out in the absence of nNOS or in the presence of EGTA, which would chelate calcium needed for CaM binding to nNOS. As shown in Figure 4*A*, when blotting for CaM the omission of nNOS from the crosslinking reaction mixture gives no CaM in the two higher molecular mass bands (*cf. lane 4 versus lane 2*). This is consistent with crosslinking of CaM to nNOS and rules out non-specific CaM crosslinking to itself to form high molecular mass bands. Moreover, EGTA caused a concentration-dependent decrease in the amount of CaM found in the higher molecular mass region (*lanes 4–6*). As a control, we verified that EGTA has no effect on the amount of nNOS present (Fig. 4*B*, *lanes 4–6*). This is further verified by quantitation of the respective bands with no change due to EGTA in nNOS (solid bars) but a large decrease in CaM bound (dashed bars). Thus, the two major bands visualized by anti-CaM are due to crosslinking of the CaM bound to nNOS in the active state. Due to the larger amounts of CaM associated with the dimeric nNOS (*D + C*), we chose to further investigate this crosslinked product for MS studies described below. In particular, we have excised the entire band corresponding to D + C on Figure 4 lane four for MS studies.

### High-resolution tandem mass spectrometric analysis of the band corresponding to nNOS dimer crosslinked to CaM

**Figure 5:**
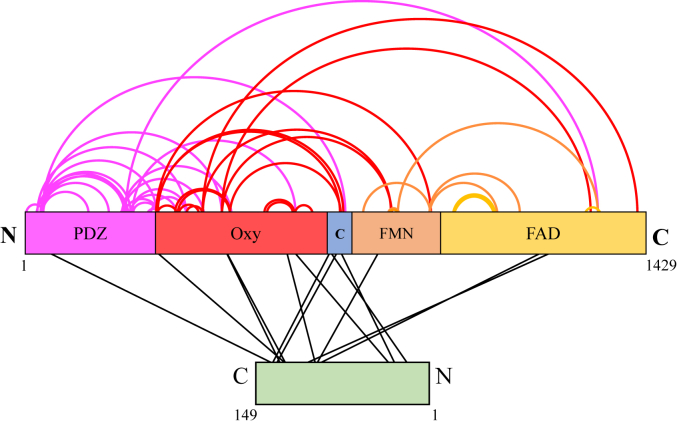
**Map of the crosslinks identified in the CaM-bound nNOS complex.** Crosslinks within the nNOS protein as well as those bridging nNOS and CaM are depicted on a linear representation of each protein. N and C terminals of each protein are labeled; PDZ, PZD domain residues 1 to 299; Oxy, heme-containing oxygenase domain residues 300 to 716; C, CaM-binding domain residues 717 to 754; FMN, FMN domain residues 755 to 1023; FAD, FAD domain residues 1024 to 1429. CaM is colored *green*. CaM, calmodulin; nNOS, neuronal nitric oxide synthase.

The band corresponding to crosslinked nNOS dimer bound to CaM described above was excised for analysis by mass spectrometry to identify the sites of crosslinking as described in Experimental procedures. In this analysis, we found 13 adducts comprising a CaM tryptic peptide crosslinked by DBSU to a nNOS tryptic peptide (Table 1). These peptides firmly establish the existence of crosslinks between CaM and nNOS in the gel band we identified by Western blotting. Furthermore, as shown in Figure 5, the 13 crosslinks that bridge the CaM and nNOS were mapped on to a linear representation of the sequence of each protein (*lines*). As expected, four crosslinks were identified between CaM and the CaM binding domain on nNOS (blue box). Interestingly, six crosslinks were found to the oxygenase domain of nNOS, and the remaining three crosslinks were found to the reductase domain of nNOS. A larger number of crosslinks were identified that are products of nNOS tryptic peptide crosslinked to nNOS tryptic peptide (Table 2). A total of 61 residues were discovered that map to various regions on the schematic shown in Figure 5 (*arcs*). It should be noted that since the nNOS is a dimer that both intra- and inter-monomer crosslinks are possible interpretations of these crosslinks, except for the case of crosslink #20, which crosslinks residue 245 of one monomer to residue 245 of the other monomer of nNOS. We will focus on the nNOS-CaM crosslinks first before examining the nNOS-nNOS crosslinks as described below.

**Table 1:** Crosslinks identified between CaM and nNOS following treatment with DSBU crosslinker

#	CaM residue	nNOS residue	CaM peptide	nNOS peptide	m/z
1	K22	K725	EAFSLFD**K**DGDGTITTK	GTNGTPT**K**R	743.869
2	T30	K733	EAFSLFDKDGDGTIT**T**K	**K**LAEAVK	700.371
3	T35	K613	DGDGTITTKELG**T**VMR	**K**MDLDMR	943.779
4	K95	Y604	EAFRVFD**K**DGNGYISAAELR	**Y**NILEEVAK	883.697
5	K95	K771	VFD**K**DGDGYISAAELR	SQAYA**K**TLCEIFK	878.193
6	K95	S1077	VFD**K**DGDGYISAAELR	NTALGVI**S**NWKDESR	910.712
7	Y100	K1080	VFDKDGDG**Y**ISAAELR	NTALGVISNW**K**DESR	911.207
8	T111	K302	HVM**T**NLGEKLTDEEVDEMIR	FL**K**VK	797.916
9	T111	K469	HVM**T**NLGEKLTDEEVDEMIR	TDG**K**HDFR	706.943
10	K116	K469	HVMTNLGE**K**LTDEEVDEMIR	TDG**K**HDFR	883.176
11	K116	K725	HVMTNLGE**K**LTDEEVDEMIR	GTNGTPT**K**R	872.680
12	T118	K38	L**T**DEEVDEMIR	ERVS**K**PPVIISDLIR	1089.578
13	T118	K725	HVMTDLGEKL**T**DEEVDEMIR	GTNGTPT**K**R	872.680

The precursor charge and identification score of each peptide are listed in Table S1. Bold underlined letters are sites of crosslinking identified by MS.

**Table 2:** Crosslinks identified within nNOS following DSBU treatment

#	Residue 1	Residue 2	Peptide 1	Peptide 2	m/z
1	aM1	K38	aMEENTFGVQQIQPNVISVR	VS**K**PPVIISDLIR	956.023
2	K24	K229	**K**VGGLGFLVK	AEM**K**DTGIQVDR	644.606
3	K24	K242	**K**VGGLGFLVK	DLDG**K**SHK	528.803
4	K24	S368	**K**VGGLGFLVK	EFLDQYYS**S**IKR	691.381
5	K33	S140	VGGLGFLV**K**ER	AVDLSHQP**S**ASKDQSLAVDR	699.577
6	K33	K188	VGGLGFLV**K**ER	ST**K**ANLQDIGEHDELLK	657.159
7	K33	K225	VGGLGFLV**K**ER	GGPA**K**AEMK	565.316
8	K33	S243	VGGLGFLV**K**ER	**S**HKAPPLGGDNDR	684.118
9	K33	K302	VGGLGFLV**K**ER	FL**K**VK	501.808
10	K33	K452	VGGLGFLV**K**ER	YATN**K**GNLR	602.339
11	K33	K732	VGGLGFLV**K**ER	AIGF**K**K	509.055
12	S215	K229	EIEPVLSILNSG**S**KATNR	AEM**K**DTGIQVDR	872.208
13	K225	K302	GGPA**K**AEMK	FL**K**VK	430.249
14	K225	K469	GGPA**K**AEMK	TDG**K**HDFR	515.509
15	K229	S243	AEM**K**DTGIQVDR	**S**HKAPPLGGDNDR	585.091
16	K229	K406	AEM**K**DTGIQVDR	DTELIYGA**K**HAWR	780.144
17	K229	K1320	AEM**K**DTGIQVDR	EPDRP**K**K	607.565
18	K242	S367	DLDG**K**SHK	EFLDQYY**S**SIKR	661.586
19	S243	K245	**S**HKAPPLGGDNDR	DLDGKSH**K**APPLGGDNDR	690.942
20	K245	K245	SH**K**APPLGGDNDR	SH**K**APPLGGDNDR	731.364
21	K245	K302	SH**K**APPLGGDNDR	FL**K**VK	439.443
22	K245	K344	SH**K**APPLGGDNDR	**K**PEDVR	576.294
23	K245	K370	SH**K**APPLGGDNDR	EFLDQYYSSI**K**R	622.511
24	K245	K620	SH**K**APPLGGDNDR	**K**TSSLWK	602.817
25	S280	K302	EQ**S**PTSGK	FL**K**VK	416.484
26	T282	K302	EQSP**T**SGK	FL**K**VK	554.976
27	K285	T289	EQSPTSG**K**QSPTK	EQSPTSGKQSP**T**K	736.87
28	S287	K302	EQSPTSGKQ**S**PTK	FL**K**VK	735.406
29	T289	K302	EQSPTSGKQSP**T**K	FL**K**VK	441.647
30	T289	K370	EQSPTSGKQSP**T**K	EFLDQYYSSI**K**R	780.393
31	K290	K302	QSPT**K**NGSPSR	FL**K**VK	497.781
32	S295	K302	NGSP**S**R	FL**K**VK	483.27
33	K302	K344	FL**K**VK	**K**PEDVR	393.986
34	K302	T724	FL**K**VK	GTNGTP**T**KR	588.003
35	K302	K725	FL**K**VK	GTNGTPT**K**R	441.253
36	K302	K733	FL**K**VK	**K**LAEAVK	530.002
37	K302	K932	FL**K**VK	TWA**K**K	488.3
38	K344	K469	**K**PEDVR	TDG**K**HDFR	479.244
39	K351	Y394	T**K**DQLFPLAK	LEEVNKEIESTST**Y**QLK	842.701
40	K351	K469	T**K**DQLFPLAK	TDG**K**HDFR	467.05
41	K370	K406	EFLDQYYSSI**K**R	DTELIYGA**K**HAWR	826.67
42	K406	K842	DTELIYGA**K**HAWR	SY**K**VR	602.573
43	K406	S1410	DTELIYGA**K**HAWR	LR**S**ESIAFIEESK	816.675
44	K452	K842	YATN**K**GNLR	SY**K**VR	471.759
45	K452	K1302	YATN**K**GNLR	N**K**GVFR	488.77
46	K469	K725	TDG**K**HDFR	GTNGTPT**K**R	701.682
47	K550	K620	HP**K**FDWFK	**K**TSSLWK	538.037
48	K555	K613	FDWF**K**DLGLK	**K**MDLDMR	593.802
49	K612	K620	YNILEEVA**K**K	**K**TSSLWK	563.565
50	K620	K660	**K**TSSLWK	VTIVDHHSATESFI**K**HMENEYR	738.373
51	T724	K732	GTNGTP**T**KR	AIGF**K**K	597.667
52	K725	K733	GTNGTPT**K**R	**K**LAEAVK	629.352
53	K778	K932	TLCEIF**K**HAFDAK	TWA**K**K	602.818
54	S833	K856	HPN**S**VQEER	**K**SSGDGPDLR	581.284
55	K842	S857	SY**K**VR	**S**SGDGPDLR	584.299
56	K856	K1320	**K**SSGDGPDLR	EPDRP**K**K	524.775
57	K932	S1083	TWA**K**K	NTALGVISNWKDE**S**R	840.112
58	K932	Y1135	TWA**K**K	LLVLSKGLQE**Y**EEWK	666.869
59	K989	S1077	LTYVAEAPDLTQGLSNVH**K**K	NTALGVI**S**NWKDESR	814.83
60	K989	S1083	LTYVAEAPDLTQGLSNVH**K**K	NTALGVISNWKDE**S**R	814.629
61	Y1292	K1321	IDHI**Y**REETLQAK	**K**YVQDVLQEQLAESVYR	1293.658

The precursor charge and identification score of each peptide are listed in Table S2.

a *Crosslink formed to N-terminal amine of residue M1*.

### Analysis of crosslinks between CaM and nNOS with the use a previously published structural model of the nNOS homodimer bound to CaM

**Figure 6:**
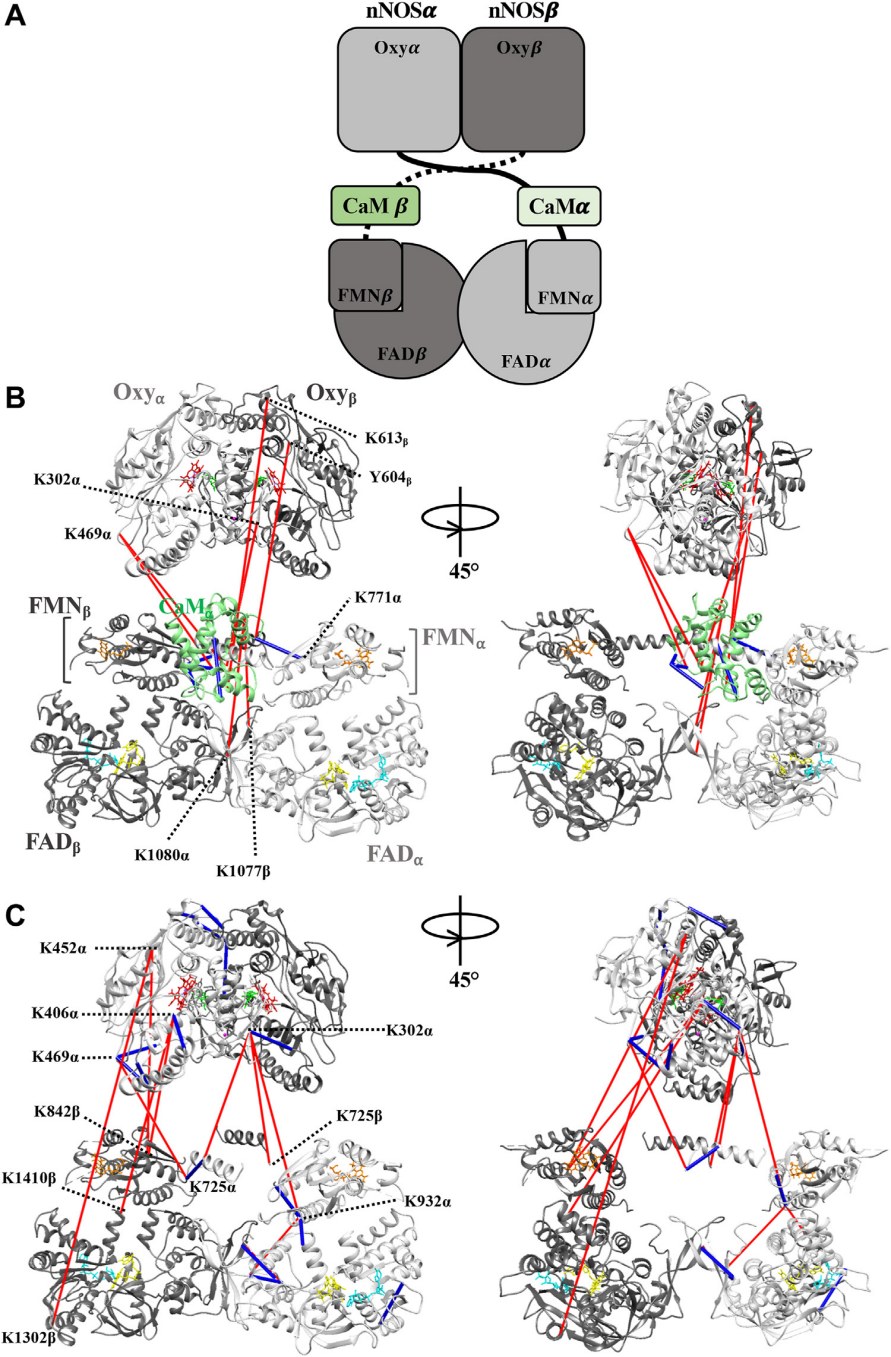
**Crosslinks mapped to EM-derived structural model of CaM-bound nNOS homodimer.***A*, schematic of the overall architecture of the CaM-bound nNOS homodimer. The nNOS homodimer is made up of monomer ⍺ (*light gray*) and monomer β (*dark gray*). There is one bound CaM (*green*) per monomer. *B*, CaM-nNOS crosslinks mapped onto the published structural model of the CaM-bound nNOS homodimer. The structure is as depicted in (*A*). Only the shortest crosslinks between CaMα (*green*) and nNOS are mapped for simplicity. The actual residues and distances are shown in Table 3. Crosslinks that meet (*blue*) or exceed (*red*) the distance constraints of the DSBU crosslinker are shown (>27 Å). *C*, crosslinks entirely within nNOS mapped to the published structural model of the CaM-bound nNOS homodimer. For simplicity, the CaM was removed and we only show the shortest crosslinks originating from monomer α as shown in Table 4. Crosslinks that meet (*blue*) or exceed (*red*) the distance constraints of the DSBU crosslinker are shown (>27 Å). Heme (*red*), BH_4_ (*green*), FMN (*orange*), FAD (*yellow*), and NADP^+^ (*blue*). CaM, calmodulin; CaM⍺, CaM bound to nNOS monomer ⍺; FAD⍺, FAD domain of nNOS monomer ⍺; FAD β, FAD domain of nNOS monomer β; FMN⍺, FMN domain of nNOS monomer ⍺; FMNβ, FMN domain of nNOS monomer β; nNOS, neuronal nitric oxide synthase; Oxy⍺, oxygenase domain of nNOS monomer ⍺; Oxyβ, oxygenase domain of nNOS monomer β.

To examine CaM-nNOS crosslinks we first mapped their positions using a structural model of the nNOS-CaM complex developed from a previously determined low-resolution negative-stain EM structure (see Experimental procedures) (6). While interpretation of the crosslink distances from this model is limited due to the resolution of the negative-stain data, this served as an important starting point for understanding the nNOS crosslink data based on domain positions. The model contains crystal structures of individual nNOS oxygenase and reductase domains as well as CaM that were docked into a low-resolution EM density map of the CaM-bound nNOS homodimer (6). As illustrated in Figure 6*A*, the functional complex of nNOS exists as a homodimer that we have arbitrarily labeled nNOS⍺ and nNOSβ with each nNOS monomer bound to its own CaM, which we have labeled CaM⍺ and CaMβ. Although each nNOS is bound to CaM, the CaM could potentially crosslink to the nNOS it is bound to or to the neighboring nNOS. Thus, for each CaM-nNOS crosslink that we identified by mass spectrometry there are four potential orientations for the crosslink based on the structure. As shown in Table 3, we measured the C_α_-C_α_ Euclidean distance of these four possible configurations of each CaM-nNOS crosslink as either CaMα to nNOSα (C⍺-N⍺) or CaMα to nNOSβ (C⍺-Nβ) or CaMβ to nNOSα (Cβ-N⍺) or CaMβ to nNOSβ (Cβ-Nβ). The values in bold are those values under the 27 Å distance restraint for the DSBU linker arm, suggesting these are plausible crosslinks. All five crosslinks (#3,4,8–10) from CaM to nNOS oxygenase domain were well above the 27 Å restraint, irrespective of the configuration. There were three crosslinks to the reductase domain (#5–7) of which only one to the FMN domain residue K771 was within the distance constraint. However, all four crosslinks between CaM and the CaM binding site identified on nNOS were well within the distance constraints (#1,2,11,13) whether the crosslink started on CaMα or for CaMβ.

**Table 3:** Cα-Cα Euclidean distances of CaM-nNOS crosslinks mapped in each possible configuration to the EM-based model of the CaM-bound nNOS homodimer

Crosslink identity					CaM-nNOS crosslink configuration			
#	CaM residue	CaM lobe	nNOS residue	nNOS domain	C⍺-N⍺	C⍺-Nβ	Cβ-Nβ	Cβ-N⍺
					C⍺-C⍺ distance (Å)			
1	K22	N-Lobe	K725	CaM helix	**14.7**	34.0	**14.7**	34.0
2	T30	N-Lobe	K733	CaM helix	**21.3**	45.6	**21.3**	45.6
3	T35	N-Lobe	K613	Oxygenase	100.1	97.9	100.2	97.5
4	K95	C-Lobe	Y604	Oxygenase	72.9	67.7	73.1	67.2
5	K95	C-Lobe	K771	FMN	**16.1**	38.7	**16.1**	38.7
6	K95	C-Lobe	S1077	FAD	34.0	33.7	34.0	33.7
7	Y100	C-Lobe	K1080	FAD	46.2	47.1	46.2	47.1
8	T111	C-Lobe	K302	Oxygenase	53.2	54.5	52.5	55.3
9	T111	C-Lobe	K469	Oxygenase	56.4	61.2	58.0	59.7
10	K116	C-Lobe	K469	Oxygenase	54.9	63.7	56.5	62.2
11	K116	C-Lobe	K725	CaM helix	**11.8**	**23.0**	**11.8**	**23.0**
12	T118	C-Lobe	K38	PDZ	NS	NS	NS	NS
13	T118	C-Lobe	K725	CaM helix	**13.4**	**25.3**	**13.4**	**25.3**

Crosslinks that satisfied the DSBU distance constraint are shown in **bold**. Crosslinks are displayed in Figure 6*B* in the orientation underlined.

Abbreviations: C, CaM; N, NOS; NS, No structural information available for one or both residues.

Although the structure of the complex is not strictly symmetrical, the distances between CaM residues to the NOS are highly similar irrespective of which CaM we chose. Thus, to simplify the visualization of the crosslinks, we focused on only those crosslinks starting from CaMα with the shortest distances to the NOS (Table 3, underlined values). These crosslinks are mapped onto the structural model shown in Figure 6*B*. The crosslinks within the accepted 27 Å distance restraint for the DSBU linker arm were mapped in blue, while those that exceeded this cutoff were mapped in red. Thus, as shown the crosslinks of CaM and the CaM-binding helix on nNOS are entirely consistent with the structural model. However, seven out of the total 13 crosslinks bridging CaM to other domains of nNOS are not consistent with the current EM-derived structural model and point to a structure where the CaM is in closer contact with the nNOS oxygenase domain.

### Analysis of nNOS-to-nNOS crosslinks with the use of the previously published structural model of CaM bound to the nNOS homodimer

Our overall approach was to take crosslinks between nNOS residues and map them onto a previously published structural model (6) to determine the C_α_-C_α_ Euclidean distances for each crosslink. In the case of nNOS crosslinks, there is an added complication in that the crosslinks could be either intra- or inter-monomer crosslinks. Thus, we needed to calculate the distances for all four possible crosslink scenarios. This is a method we used to characterize the inter- and intra-monomer crosslinks found for the homodimer of CYP102A1 (14). Although we identified 61 crosslinks between nNOS residues, 32 of these crosslinks involved residues in the PDZ domain, where structural information is lacking, and no corresponding EM densities were established. As shown in Table 4, we sorted the 29 remaining crosslinks by those that involve crosslinks between oxygenase domain residues (*Oxygenase-Oxygenase*), between CaM binding domains (*CaM Helix-CaM Helix*), between reductase domain residues (*Reductase-Reductase*), and those that crosslink between oxygenase and reductase domain residues (*Interdomain*).

**Table 4:** Cα-Cα Euclidean distances of crosslinks between residues of nNOS mapped in each possible configuration of the EM-based model of the CaM-bound nNOS homodimer

			Crosslink configuration			
#	Residue 1	Residue 2	⍺-⍺	⍺-β	β-β	β-⍺
**Oxygenase-oxygenase**			**C⍺-C⍺ distance (Å)**			
33	K302	K344	35.1	**19.8**	33.7	**18.2**
38	K344	K469	**16.8**	60.5	**17.4**	58.9
39	K351	Y394	**10.0**	62.5	**10.5**	62.2
40	K351	K469	**16.0**	67.1	**17.9**	65.5
41	K370	K406	**17.6**	60.1	**17.4**	60.0
47	K550	K620	**25.7**	**16.2**	**25.6**	**16.2**
48	K555	K613	**11.5**	28.4	**11.7**	28.5
49	K612	K620	**13.8**	28.0	**13.4**	28.1
50	K620	K660	40.8	**19.4**	40.6	**19.4**
**CaM binding domain-CaM binding domain**						
51	T724 *(C-Binding)*	K732 *(C-Binding)*	NS	NS	NS	NS
52	K725 *(C-Binding)*	K733 *(C-Binding)*	**18.6**	35.5	**18.6**	35.5
**Reductase-Reductase**						
53	K778	K932	**13.3**	57.1	**13.3**	57.1
54	S833	K856	a**19.3**	NS	a**19.3**	NS
55	K842	S857	a**25.6**	NS	a**25.6**	NS
56	K856	K1320	NS	NS	NS	NS
57	K932	S1083	32.2	44.7	32.2	44.7
58	K932	Y1135	**12**	66.6	**12**	66.6
59	K989	S1077	28.1	**16.2**	28.1	**16.2**
60	K989	S1083	**12**	**25.5**	**12**	**25.5**
61	Y1292	K1321	**19.3**	111.4	**19.3**	111.4
**Interdomain**						
34	K302 *(Oxy)*	T724 *(C-Binding)*	NS	NS	NS	NS
35	K302 *(Oxy)*	K725 *(C-Binding)*	61.9	58.1	61.4	59.1
36	K302 *(Oxy)*	K733 *(C-Binding)*	46.1	49.6	45.6	50.5
37	K302 *(Oxy)*	K932 *(FMN)*	61.2	74.3	60.4	73.8
42	K406 *(Oxy)*	K842 *(FMN)*	83.4	73.1	82.7	72.4
43	K406 *(Oxy)*	S1410 *(FAD)*	88.8	85.8	88.1	85.0
44	K452 *(Oxy)*	K842 *(FMN)*	104.2	85.7	103.3	85.6
45	K452 *(Oxy)*	K1302 *(FAD)*	153.8	132.0	153.0	131.6
46	K469 *(Oxy)*	K725 *(C-Binding)*	52.5	72.7	54.1	74.2

Crosslinks that satisfied the DSBU distance constraint are shown in **bold**. Crosslinks are displayed in Figure 6*C* in the orientation underlined. NS, No structural information available for one or both residues.

Abbreviations: C-Binding, CaM-binding domain; FAD, FAD-binding domain; FMN, FMN-binding domain; Oxy, oxygenase domain.

a Structural information only available in crystal structure 1TLL, not resolved in EM-based reductase dimer.

#### Crosslinks within the nNOS oxygenase domain

We found nine crosslinks that are derived from residues entirely in the nNOS oxygenase domain (Table 4, *Oxygenase-Oxygenase*). In six of these cases (#38–41, 48, 49), the intramonomer crosslinks are shorter and fall within the 27 Å distance cutoff for the DSBU linker arm. For two of the crosslinks (#33, 50) only the intermonomer crosslinks are within the appropriate distance. In one case (#47), the crosslink distance for inter- and intra-monomer crosslinking are both under the distant constraint, although the intermonomer crosslink is shorter. Due to the near symmetry of the nNOS oxygenase dimer, the intramonomer crosslinks are not that different in either the nNOSα or nNOSβ. Similarly, the intermonomer crosslinks do not differ greatly whether they originate from nNOSα or nNOSβ. In all the crosslinks within the oxygenase domain, the structure is consistent with the structural constraints of the crosslinker.

#### Crosslinks within the CaM binding domain

There are two crosslinks found in the CaM binding domain that crosslink lysine residue pairs very close to each other. Only one of these crosslink pairs (#52) resided in the defined areas of the structural model and was found to bridge residues in our structural model within the distance constraint of the crosslinker.

#### Crosslinks within the nNOS reductase domain

We found nine crosslinks derived from residues entirely within the nNOS reductase domain (Table 4, *Reductase-Reductase)*. In seven of these crosslinks, a conformation exists that fits the distance constraints of the crosslinker and is thus consistent with the structural model. Unlike the crosslinks found for the oxygenase domain, one of the crosslinks (#57) did not fit the structure and an additional crosslink (#56) has no structural information available for the residues involved. However, there seems to be a good fit overall for the reductase domain crosslinks to the available structure.

#### Crosslinks that span the nNOS oxygenase and nNOS reductase domains

There are nine crosslinks that span from the oxygenase to the reductase domains of nNOS (Table 4, *Interdomain*). One of these crosslinks (#34) involves residues where the structures are not resolved from the available studies. The remaining eight interdomain crosslinks (#35–37, #42–46) did not fit the existing structure with distances far exceeding the cutoff for the crosslinker. Thus, these crosslinks between the domains do not fit well to the available structure of the full length nNOS.

#### Visualizing the nNOS-to-nNOS crosslinks

Based on our analysis of the distances between residues in the four possible scenarios outlined above, we chose to map only those crosslinks originating from nNOSα and spanning to a residue with the shortest distance irrespective of whether they were intra or inter-protein crosslinks (Table 4 crosslinks with underlined distance values). This greatly simplified the visualization of the crosslinks. As shown in Figure 6*C*, these crosslinks were mapped onto the EM derived structural model of the full-length nNOS homodimer, which we used above for visualizing the CaM-based crosslinks. The nNOS-nNOS crosslinks that satisfied the distance constraints of the DBSU crosslinker are shown in blue and those that exceed the 27 Å cutoff are shown in red. Thus, we can clearly see that crosslinks within the oxygenase or reductase domains are readily mapped within the distance constraints whereas all the crosslinks that span two domains from oxygenase to reductase or oxygenase to CaM-binding domain clearly are longer distances than expected from the structural model. Although the full structure of the CaM-bound nNOS homodimer is not symmetric, the analogous crosslinks originating from nNOSβ, which were not mapped, provide a highly similar picture of the crosslinks to that shown here. Although the published model served as a key starting point for 3D analysis of our crosslink data, the crosslink distances are too long (>45 Å) at many sites in the map (see Table 4, Fig. 6) for the expected Cα-Cα distance of a DSBU crosslink (<27 Å) (11, 12, 15). These discrepancies indicate the nNOS dimer may adopt different conformations in our crosslinked sample compared to the published negative-stain model (4). Taken together the CaM-nNOS and nNOS-nNOS crosslinks clearly point to an architecture of nNOS where the oxygenase and reductase domains are closer together. Thus, we pursued cryo-EM analysis of the uncrosslinked and crosslinked CaM-bound nNOS homodimers in order to obtain an improved model that better matches the crosslink data.

### Cryo-EM studies of uncrosslinked and DSBU-crosslinked CaM-bound nNOS homodimer

**Figure 7:**
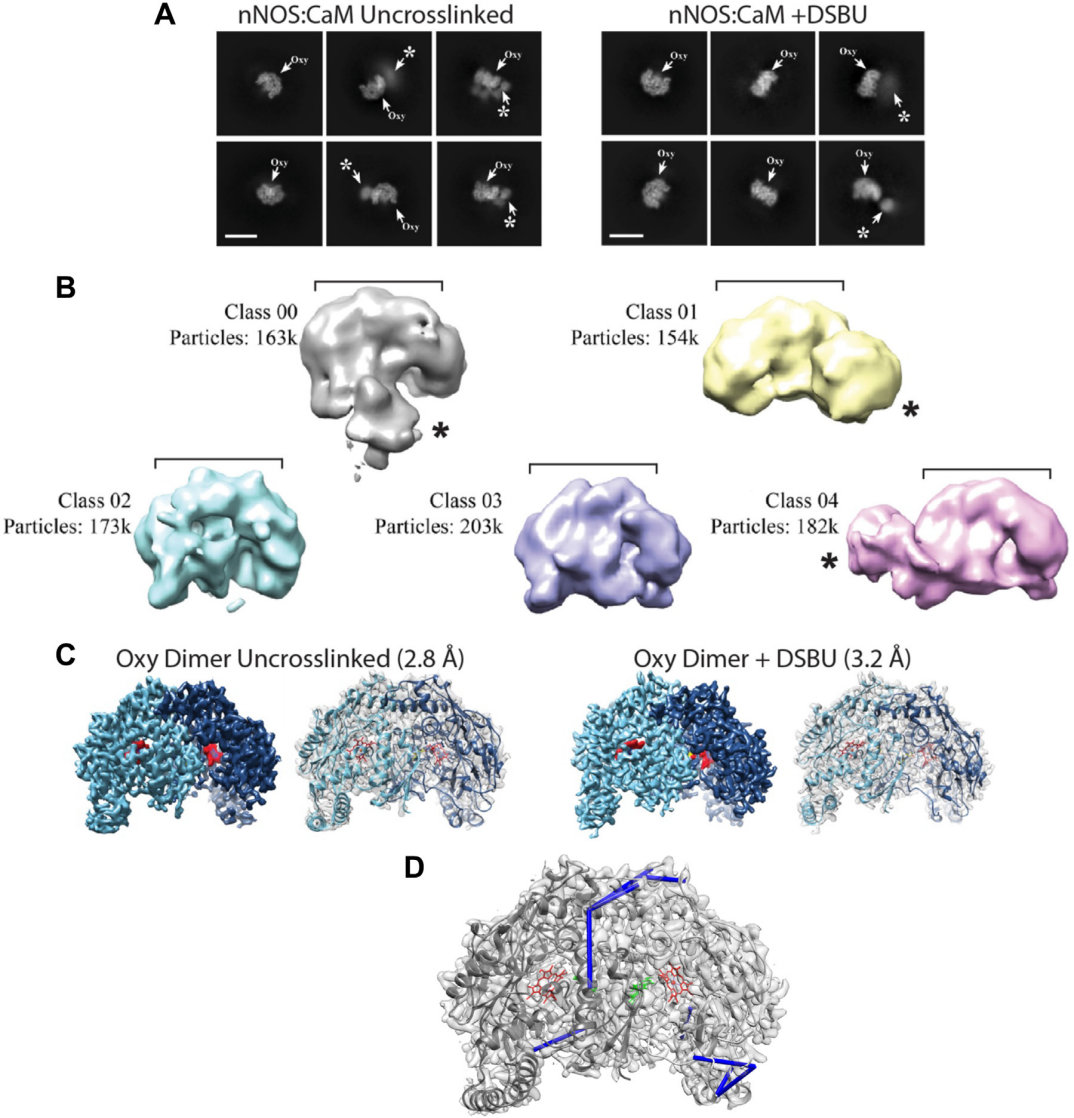
**Cryo-EM analysis shows high-resolution oxygenase dimer and poorly resolved reductase domain for uncrosslinked and DSBU-crosslinked nNOS:CaM.***A*, reference-free cryo-EM 2D class averages of uncrosslinked (*left*) and DSBU-crosslinked (*right*) nNOS:CaM showing well-resolved central density for the oxygenase dimer (Oxy) in all classes and diffuse additional densities in certain classes (∗). Scale bar represents 100 Å. *B*, cryo-EM 3D classification of uncrosslinked nNOS:CaM showing density for oxygenase dimer (*brackets*) and adjacent globular densities in classes 00, 01, and 04. *C*, refined cryo-EM map (*left*) and docked model (*right*) of the oxygenase (Oxy) dimer for the uncrosslinked and DSBU-crosslinked nNOS:CaM samples at 2.73 Å and 3.14 Å resolution, respectively, colored to show oxygenase monomers (*light* and *dark blue*) and heme cofactor (*red*). *D*, oxygenase-oxygenase crosslinks (*blue* lines) shown on the DSBU-crosslinked nNOS:CaM cryo-EM model. The crosslinks shown are listed on Table 4 as oxygenase-oxygenase crosslinks. The shortest orientations of the crosslinks, which are underlined on Table 4, are shown. CaM, calmodulin; nNOS, neuronal nitric oxide synthase.

Given the potential limitations of the published structural model used above for verifying the positions of the crosslinks we next sought to characterize uncrosslinked and DSBU-crosslinked CaM-bound nNOS homodimer by cryo-EM. By 2D classification, both showed well-resolved density for the oxygenase dimer and diffuse additional density we postulate to be the flexibly-linked reductase domains (Fig. 7*A*). Low-resolution 3D classification analysis of uncrosslinked nNOS:CaM shows strong density for the oxygenase dimer in all classes and additional globular density adjacent the dimer in classes 00, 01, and 04 (Fig. 7*B*). Of these classes, Class 04 exhibited the clearest density for the oxygenase dimer and the largest and best-resolved additional density adjacent the dimer. Notably, 3D classification of the crosslinked sample did not resolve additional densities beyond the oxygenase. This could be due to DSBU crosslinking causing an increased heterogeneity in the position of the reductase domain that impacts the 3D alignment. We also see fewer additional densities in the 2D analysis of the crosslinked sample compared to uncrosslinked (Fig. 7*A*), further indicating there is an increase in heterogeneity of the reductase domain. The oxygenase dimer for both the uncrosslinked and crosslinked samples resolved to high-resolution by 3D refinement, enabling an atomic model to be built (Figs. 7*C* and S1, *A*–*H*). However, no additional density corresponding to the reductase domains could be resolved to high resolution for either map. Focused refinements using a mask around the extra globular densities were tested in an attempt to improve resolution for this region. However, no improvements were obtained, indicating the flexibility, potentially combined with the smaller size of this region, continued to impair refinements. The oxygenase dimer structures are nearly identical for both uncrosslinked and crosslinked nNOS:CaM (Cα RMSD = 0.3 Å) as well as for a previously published crystal structure (Cα RMSD = 0.8 Å for PDB: 1ZVL), indicating that crosslinking induces no detectable structural changes. Additionally, we have mapped the oxygenase-oxygenase crosslinks to the structure (Fig. 7*D*). These exhibit short distances (see Table 4) and are in agreement with our molecular model for the oxygenase dimer.

### Crosslink-guided intermolecular docking of CaM and the FMN domain to nNOS

**Figure 8:**
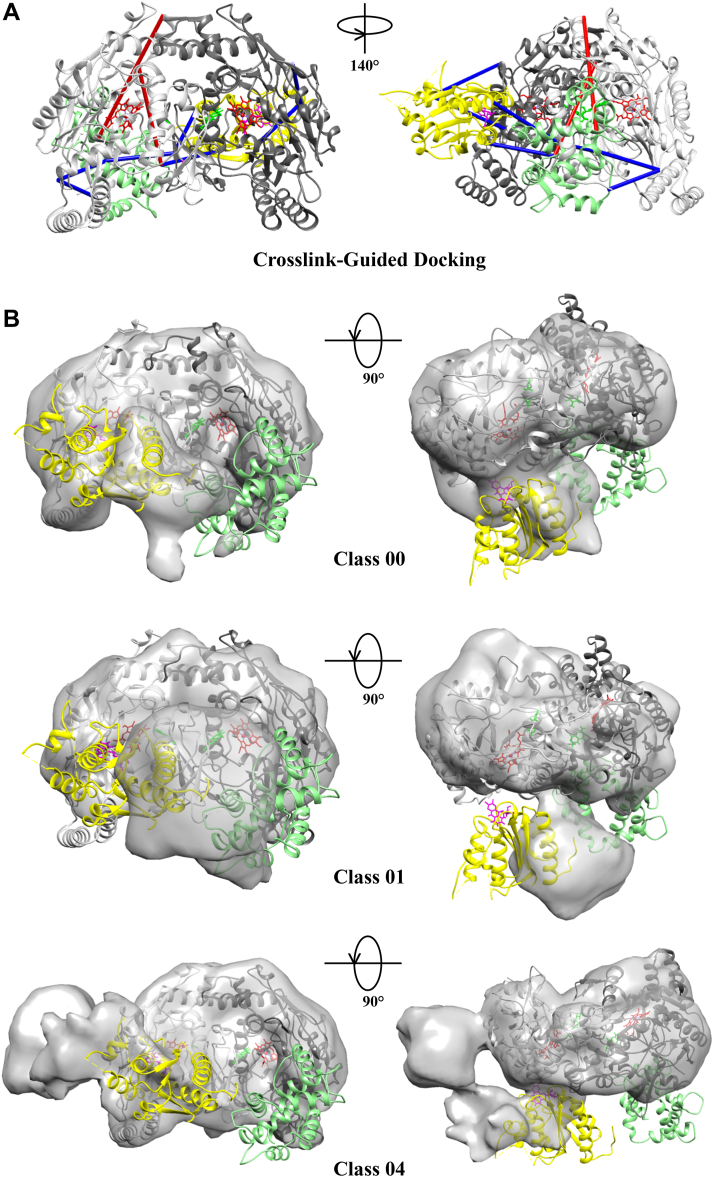
**Structural model of CaM-bound nNOS homodimer generated with the use of crosslink-guided docking and fitting into cryo-EM densities.***A*, crosslink-guided docking of CaM_α_ and nNOS FMN_α_ to the nNOS oxygenase domain dimer. The nine *red* crosslinks, which represent all the crosslinks observed between these domains, were used to guide the docking experiment using HADDOCK as described in Experimental procedures. The best scoring structure from the HADDOCK crosslink-guided docking experiment is shown. Of the original nine crosslinks that exceeded the distance constraints (>27 Å), seven now meet the distance criteria (*blue* lines) with only two exceeding the limits (*red* lines). *B*, crosslinked-derived docked model from (*A*) containing CaM_α_, nNOS FMN_α_, and nNOS oxygenase domains was fitted to cryo-EM densities from Figure 7*B*, class 00, 01, and 04. The crosslink-derived docking model was fit into the densities (*gray*) as described in Experimental procedures. CaM (*green*) and FMN domain (*yellow*) are shown. The prosthetic groups are heme (*red*), BH_4_ (*green*), and FMN (*pink*). CaM, calmodulin; nNOS, neuronal nitric oxide synthase.

As discussed above, when the crosslink sites are mapped onto the previously published nNOS:CaM model we identify distances that are longer than what is expected for DSBU, indicating oxygenase-reductase position in the model is not in good agreement with our crosslinking analysis (Table 4, Fig. 6*B*) (4). Given that a closer oxygenase-reductase position is expected for electron transfer, an alternate domain arrangement based on the DSBU crosslink sites may better support the active conformation for NO synthesis. Therefore, to visualize a conformation where the crosslinks might better fulfill the distance constraints, we utilized HADDOCK to dock only the FMN domain from the alpha monomer of nNOS and the CaM protein bound to the alpha monomer of nNOS to the entire nNOS oxygenase dimer containing the oxygenase domains from both the alpha and beta monomers of nNOS. As a minimum of three crosslinks between domains are appropriate for crosslink-guided docking analysis between domains (16), we did not include the large FAD/NADPH binding domain for the docking calculations. As shown in Table 5, the five crosslinks (#3, 4, 8–10) between CaM and nNOS oxygenase domain, in addition to three crosslinks (#37, 42, 44) between the FMN domain and the oxygenase domain, were used to guide the docking experiment. To generate a new model, the crosslinks were input as docking restraints to guide domain positioning during the docking experiment, with distance cutoffs of 3 to 27 Å. As noted in Methods, the docking was performed with 1000 rigid body docking iterations, 200 semi-flexible refinements, and 100 final refinements. From this analysis, we obtained four clusters of possible docked structures. These results had similar orientations of the CaM and FMN relative to oxygenase domain dimer with minor upwards and downwards shifts in the positioning of both CaM and FMN relative to oxygenase dimer, but no difference in the orientation of FMN domain or CaM relative to one another or rotation about the areas of surface contact. We present here the top docking result having the best HADDOCK score of −142.7 ± 25.3 with a predicted van der Waals energy of −70.0 ± 9.1 kcal/mol and a predicted electrostatic energy of −557.0 ± 57.5 kcal/mol as FMN and CaM contacted the oxygenase dimer (Fig. 8*A*). The crosslinks we used to guide the docking are shown in Table 5 with the calculated distances based on the initial negative-stain EM derived structural model (*Negative-Stain EM model*) as well as our new model derived after docking (*XL-guided* m*odel*). Although one crosslink (#5) was within the accepted distance constraint of the crosslinker even before docking, the crosslinked-guided model gave distances within 27.1 Å for all but one crosslink (#3) within the domains we used. For crosslink #3 bridging CaM to oxygenase domain, the distance was reduced from approximately 100 Å to 42 Å. We found that the three crosslinks when mapped between the FMN domain of the alpha monomer of nNOS to the oxygenase domain of the beta monomer of nNOS were all within 27.1 Å (Red⍺-NOSβ) whereas those same crosslinks mapped as intra-monomer crosslinks, from the FMN domain and the oxygenase domain of the alpha monomer were all over 55 Å (Red⍺-NOS⍺). This is consistent with the finding that the active nNOS employs a *trans* delivery of electrons from the FMN moiety of one monomer to the heme of the other monomer in the nNOS homodimer (2).

**Table 5:** Refinement of the CaM-bound nNOS model with the use of crosslink-guided fitting of reductase ⍺ and CaM⍺ onto the nNOS oxygenase domain dimer

#	Residue 1	Residue 2	Negative stain EM model		XL-guided model	
			CaM⍺-NOS⍺	CaM⍺-NOSβ	CaM⍺-NOS⍺	CaM⍺-NOSβ
	CaM⍺	nNOS⍺ or nNOSβ				
3	T35	K613 *(Oxy)*	100.1	97.9	41.7	57.5
4	K95	Y604 *(Oxy)*	72.9	67.7	**24.5**	48.1
5	K95	K771 *(FMN)*	**16.1**	38.7	**20.3**	NP
6	K95	S1077 *(FAD)*	34.0	33.7	NP	NP
7	Y100	K1080 *(FAD)*	46.2	47.1	NP	NP
8	T111	K302 *(Oxy)*	53.2	54.5	41.5	**15.1**
9	T111	K469 *(Oxy)*	56.4	61.2	**26.1**	61.4
10	K116	K469 *(Oxy)*	54.9	63.7	**25.1**	60.6


Reductase ⍺		nNOS⍺ or nNOSβ	Red⍺-NOS⍺	Red⍺-NOSβ	Red⍺-NOS⍺	Red⍺-NOSβ
37	K932 *(FMN)*	K302 *(Oxy)*	61.2	73.8	55.7	27.1
42	K842 *(FMN)*	K406 *(Oxy)*	83.4	72.4	75.1	**26.7**
43	S1410 *(NAD)*	K406 *(Oxy)*	88.8	85.8	NP	NP
44	K842 *(FMN)*	K452 *(Oxy)*	104.2	85.6	79.6	**26.9**
45	K1302 *(NAD)*	K452 *(Oxy)*	153.8	132.0	NP	NP
57	K932 *(FMN)*	S1083 *(FAD)*	32.2	44.7	NP	NP
58	K932 *(FMN)*	Y1135 *(FAD)*	**12.0**	66.6	NP	NP

Crosslinks that satisfied the DSBU distance restraint are shown in **bold**. Crosslinks are displayed in Figure 8 in the orientation underlined.

Abbreviation: NP, domain not present.

As shown in Figure 8*A* the crosslinks were visualized as they appear in the crosslink-guided model (Table 5, *underlined values, XL-guided model*). As expected, the CaM and FMN domain are in much closer proximity to the nNOS oxygenase domains after docking. The FMN moiety from the alpha monomer is seen approaching the heme moiety of the beta monomer in this depiction within 14.5 Å. Although this is only one possible configuration, these studies point to a dynamic structure for nNOS that entails greater flexibility of the reductase domain relative to the more stable oxygenase dimer. This is supported, in part, by the inability to get high resolution structures of the reductase domain in cryo-EM studies.

We next compared the top crosslink-guided model to the low resolution cryo-EM maps derived from 3D classification of uncrosslinked nNOS-CaM (Fig. 7*B*). We compared classes 0, 1, and 4, which contain additional globular density adjacent the oxygenase dimer. The crosslink-guided model was fit into each of these three classes using Chimera’s “Fit in Map” feature and are shown in Figure 8*B*. While the oxygenase dimer fit well into these maps, the FMN domain and CaM positions predicted by crosslink-guided docking did not match precisely with the additional densities in the cryo-EM maps for any of the classes. However, the FMN partially occupies or is positioned adjacent these densities in all three classes, indicating slight re-arrangements could result in better agreement with the cryo-EM maps and crosslink distances. Considering the cryo-EM data are from uncrosslinked nNOS:CaM samples, theses discrepancies could reflect differences in reductase flexibility or position due to crosslinking. Together, these data support a reductase-oxygenase orientation that is closer than what has been previously modeled and provide a plausible model for electron transfer across the FMN-oxygenase domains. However, further studies are needed to obtain a high-resolution structures of the intact complex to definitively resolve the reductase position and assign the crosslinks to the structure.

## Discussion

We utilized the MS-cleavable bifunctional crosslinking reagent DSBU and high-resolution MS to accurately map the crosslinked residues and determine the protein interaction sites of the active CaM-bound nNOS homodimer in solution. We have previously utilized this method to successfully map the protein interactions of CYP102A1 (14), which is a flavohemoprotein homodimeric enzyme with a similar architecture to nNOS (17, 18). As performed for CYP102A1, we characterized the reaction kinetics and optimized conditions for the crosslinking as well as analyzed crosslinks in all possible combinations since both intra- and inter-monomer crosslinks could be formed. We obtained 61 crosslinks within nNOS, along with 13 crosslinks between nNOS and bound CaM. The crosslinks bridging residues entirely within the nNOS oxygenase domain or the nNOS reductase domain were consistent with the high-resolution crystal structures previously reported for these domains (19, 20). Similarly, the crosslinks between CaM and the previously established CaM binding helix on the nNOS linker region that exists between the oxygenase and reductase domains were also consistent with the crystal structure of that region [PDB 2O60]. In contrast, the other crosslinks between nNOS and CaM, as well as crosslinks between oxygenase and reductase domains of nNOS, were found to exceed the maximum distance possible for DSBU. Mapping these outlier crosslinks onto the EM-derived structural model for nNOS (6) revealed that these crosslinks represented a conformational state where CaM is in closer proximity with the nNOS oxygenase domain, as well as a conformation where the flavin-containing reductase domains is closer to the heme-containing oxygenase domain. This notion is consistent with the cryo-EM and negative-stain EM studies of nNOS, iNOS, and eNOS that showed large conformational shifts of the NOS reductase domain with respect to the oxygenase domain (6, 7, 8, 21, 22). Due to the low resolution of the structures derived from the EM densities, the exact mechanism of these dynamic movements is still not clearly defined. Our results clearly point to a conformation where the reductase domain and CaM achieve a closer proximity with the oxygenase domain. In an attempt to visualize the crosslinks, cryo-EM studies were carried out on the native and crosslinked CaM-bound nNOS samples. We failed to observe any differences due to crosslinking, most likely due to the heterogeneity of the crosslinked nNOS products formed. Although we also failed to obtain a structural model of the reductase domain, we succeeded in obtaining a higher resolution 3D model of the nNOS oxygenase dimer.

To better visualize how the domains could be configured to account for the outlier crosslinks, we performed a HADDOCK-based docking experiment using crosslinks to guide the orientation of the FMN domain and CaM relative to the oxygenase domain dimer. We found that CaM localizes around the opening to the substrate cleft in the oxygenase domain, covering nNOS surface residues 297, 299 to 302, 309, 486, 488, 503, 507 to 508, 712 to 713, and 716. The docked FMN domain directly contacts the opposing oxygenase domain covering residues 368, 370, 406, 420 to 423, 660, and 667. HDX-MS studies of nNOS did not detect any CaM-induced shielding of exchange for any of these nNOS residues (10). Such shielding would be expected if either the FMN-domain or CaM were to stably bind at these protein surfaces. In fact, CaM-induced shielding of protons was only found in the CaM-binding region residues 731 to 744, as well as a linker region residues 749 to 758 where high affinity binding of CaM occurs (10). As noted by the authors, the absence of shielding in other areas could be due to the highly transient nature of these interactions or the low abundance of such conformations. Conformations involving the shuttling of electrons from the FMN to the heme are thought to be highly transient.

Interestingly, an HDX-MS study of iNOS domains clearly showed direct interactions of the FMN subdomain, heme domain, and CaM (9). In particular, shielding was observed on residues corresponding to nNOS residues 299 to 302, 309, 503, and 507 to 508 (9). These residues are predicted by our crosslinked guided docked model to be covered by interactions with CaM. In contrast to the nNOS study where the full-length protein was used, the individual FMN domain and the heme domains of iNOS was used along with CaM to perform the HDX-MS studies. Perhaps the use of the domains allowed the HDX-MS studies to more easily capture a conformation where the FMN domain was closer to the heme domain. It is likely that the untethered FMN domain has affinity for the oxygenase domain, but this is masked by the need for this domain to interact with the FAD domain for efficient shuttling of electrons from NADPH. We are not aware of any HDX-MS studies with nNOS and the use of these domains so we cannot be certain that this explains the differences amongst the finding for the HDX-MS studies. In any case, the nature of the domains on iNOS certainly better mimics the crosslink-guided docking studies performed here. Thus, these HDX-MS studies along with EM studies of iNOS provide further evidence for the interaction of the FMN domain and CaM with the oxygenase domain and strongly suggest that such interactions are likely to occur for nNOS. It appears that the crosslinking method used here may have utility in capturing very transient or low population conformations of multiprotein complexes.

As noted above, nNOS oxygenase residues 420 to 423, 660, and 667 are in contact with the FMN-domain in our docked model. It is noteworthy that mutations of residues 421, 423, 660, and 667 reduced the rate of heme reduction and subsequent NO production by nNOS (23, 24). Moreover, residue 423 has been proposed to be part of the lower “lip” of the heme access point for electron transfer in nNOS and is thought to play a critical role in the transfer of electrons (10, 25). Taken together, these studies are consistent with our docking of the FMN-domain to the oxygenase domain.

Our docking experiment also positioned the FMN domain closer to the nNOS oxygenase dimer such that FMN from one monomer was 14.5 Å away from the heme of the opposing monomer whereas it was 45.2 Å away from the heme of the same monomer. This finding of the shorter distance to the neighboring monomer’s heme is consistent with the biochemical evidence that established the *trans* electron flow from the FMN of one monomer to the heme of the opposing monomer (26). While we do not propose the docked structure acquired here is the precise electron transfer configuration of the oxygenase-FMN complex, it represents a conformation of the nNOS with FMN closer to heme than in the conformation captured previously by EM (62.0 Å). While this model provides further evidence for a suite of nNOS conformations, additional studies will be required to determine the precise orientation of FMN and heme during the critical step of electron transfer. These results indicate that nNOS, eNOS, and iNOS may have a common mechanism involving interactions of the FMN domain with the oxygenase domain that is facilitated by CaM interactions with these domains. Our findings certainly point to the great utility of crosslinking to discover highly transient conformations in complex multiprotein enzyme systems. However, a caveat with all crosslinking studies is the potential for non-specific crosslinking reactions although we have thoughtfully tried to minimize these reactions.

Our modeling studies are consistent with all the crosslinks found, however a higher resolution structure is needed to validate this model and determine the precise configuration of nNOS that allows electron transfer to heme. Unfortunately this has eluded the field although we have taken a significant step by resolving the oxygenase dimer at higher resolution. The need for a higher-resolution full-length structure is especially true with the long-range crosslinks found for residues in the PDZ domain. The validation of these crosslinks will provide novel insights on PDZ domain localization and potential regulatory mechanisms of this relatively poorly understood component of nNOS.

## Experimental procedures

### Materials

DSBU (Lot UG281415) was purchased from Thermo Fisher Scientific. Sodium chloride (Lot 16620) was from Fisher BioReagents. Anhydrous DMSO (Lot SHBK9388), 2-mercaptoethanol (Lot SHBG9616V), Hepes (Lot SLBW4677), ammonium bicarbonate (Lot 116K0130), and rabbit anti-nNOS antibody (N7155) were from Sigma. The 10x Tris/Glycine/SDS (Lot 64343645), Bio-Safe Coomassie stain (Ctrl BR03262020), 2x Laemmli (Lot 64315141), and 4 to 15% gradient SDS-PAGE gels (Lot 64362663) were purchased from Bio-Rad. Mouse anti-CaM antibody was from Millipore (05-173, Lot 2829830). Goat anti-rabbit (926–32211) and goat anti-mouse (926–68020) infrared dye-conjugated antibodies were from Li-Cor.

### Protein expression and purification

Full-length rat nNOS was expressed and purified as previously described (27). The purification entailed affinity chromatography on an ADP-Sepharose column and subsequent chromatography on a Mono Q ion exchange column. The purified nNOS had a specific activity of 504 nmol NO/min/mg as assessed by the oxyhemoglobin method described below. The purity of the nNOS was determined by SDS-PAGE, Coomassie staining, and densiometric analysis to be at least 92%. The expression vector for human CaM containing an N-terminal His_6_-TEV tag was generously provided by John Tesmer (Purdue University). Human CaM was overexpressed in *E. coli*, purified by nickel agarose chromatography, and treated with TEV protease to remove the tag (28). For cryo-EM studies, non-tagged human CaM was expressed in *E. coli* and purified by hydrophobic interaction chromatography with phenyl Sepharose resin as described previously (6). The human CaM protein has an identical protein sequence to the rat CaM. The CaM purity was determined by Coomassie staining of gels to be at least 93%.

### nNOS activity

NO synthesis was determined by measuring the NO-catalyzed conversion of oxyhemoglobin to methemoglobin (29). The nNOS (1.3 μg) was incubated with 100 μM CaCl_2_, 100 μM L-arginine, 100 μM tetrahydrobiopterin, 100 units/ml catalase, 25 μM oxyhemoglobin, and an NADPH-regenerating system consisting of 400 μM NADP^+^, 10 mM glucose 6-phosphate, and 1 unit/ml glucose-6-phosphate dehydrogenase, expressed as final concentrations, in a total volume of 180 μl of 50 mM Tris, pH 7.4. at 37 °C. The rate of oxidation of oxyhemoglobin was measured with a microtiter plate reader as described previously (27, 29, 30).

### Chemical crosslinking, SDS-PAGE, and Western blotting

Purified nNOS (2 μM) was mixed with CaM (2.4 μM), 10 μM CaCl_2_, 10 μM tetrahydrobiopterin, and 0.1 mM NaCl in total volume of 10 μl of Hepes, pH 7.35 for 5 min at 4 °C. The mixture was treated with the desired amount of DSBU, which was dissolved in anhydrous DMSO, at room temperature with rotation. The DMSO concentration was 10% (v/v) of the total reaction mixture. As a control, we showed that 10% DMSO had no effect on the CaM-dependent NOS activity. Reactions were quenched at the appropriate times with 2 μl of 0.5 M ammonium bicarbonate and gently mixed for 5 min at 4 °C before addition of an equal volume of 2x Laemmli sample buffer containing 10% (v/v) 2-mercapotethanol. Samples were boiled for 5 min, and aliquots (15 μl) were submitted to SDS-PAGE on 4 to 15% gradient gels run for 45 min at 50 mAmps/gel. Gels were Coomassie stained for 1 h with ProteinSafe Coomassie Stain and destained in MilliQ water. Gels were imaged with a LI-COR Odyssey Fc and bands corresponding to nNOS monomer and dimer were quantified by densitometric analysis with the use of ImageStudio software (version 5.2; www.licor.com/bio/image-studio/).

Aliquots (7.5 μl) of the reaction mixtures were run on SDS-PAGE as above and further analyzed by Western blotting. Gels were transferred to Immobilon-FL PVDF membranes at 100 V for 2 h in a Mini *Trans*-Blot Electrophoretic Transfer Cell (Bio-Rad). The membranes were blocked for 30 min and then incubated with anti-nNOS (0.01% v/v, Sigma N7155) or anti-CaM (1.5 μg/ml, Millipore 05-173) at room temperature for 1 h. The blots were washed and incubated with the appropriate IR-tagged secondary antibody (0.4 μg/ml anti-rabbit and 0.2 μg/ml anti-mouse) for 1 h. The IR signal for nNOS and CaM were imaged with a LI-COR Odyssey Fc.

### Mass spectrometry and peptide assignment

Crosslinked protein samples were separated by SDS-PAGE. Duplicate protein bands corresponding to CaM-bound nNOS homodimer were submitted for in-gel trypsinolysis and subsequent analysis on a Thermo Scientific Q Exactive HF Orbitrap MS at the University of Michigan Mass Spectrometry-Based Proteomics Resource Facility. Sequence coverage was determined using Proteome Discoverer (version 2.5). Coverage of CaM was 100%, and of nNOS was 96.4%. Peptide assignments were performed using MeroX (version 2.0) (31) to specifically search for peptides containing the signature doublet that DSBU produces upon fragmentation. MS datasets were analyzed with primary and secondary fragment mass deviations of 10 and 50 ppm, respectively, with mass limits of 600 to 6000 Da and s/n ratio of 1.5. Three missed cleavage sites per fragment were permitted. Score cut-offs calculated for a false discovery rate <0.01% were applied as recommended in the literature (13, 15). The .zhrm file uploaded in the PRIDE directory of the Proteome Xchange repository for this paper contains annotated secondary spectra for every hit identified by MeroX. Representative spectra are shown in Fig. S2. The mass spectrometry proteomics data along with the MS/MS data have been deposited to the ProteomeXchange Consortium *via* the PRIDE (32) partner repository with the dataset identifier PXD044750.

### Mapping of crosslinks onto three-dimensional models of nNOS

An EM-based structural model of the CaM-bound nNOS homodimer was used to guide for *in silico* model for crosslink analysis. Rigid-body fitting results of crystal structures of NOS heme, FMN, and FAD domains [PDB 1RS9, 3HR4, 1TLL] into EM density of the nNOS homodimer [EMDB 5940] (6). Crystal structures of nNOS oxygenase and FMN domains [PDB 1ZVL, 1TLL_750-951_] were aligned with the corresponding domains in this model using UCSF Chimera (Version 1.13.1) (33). An existing structure of CaM bound to the nNOS CaM-binding helix has previously been resolved [PDB 2O60]; however, the N-terminal residue K725 in the nNOS CaM-binding helix is not present in the structure. To examine crosslinks involving this residue, the CaM-binding helix of iNOS [PDB 3GOF] was sequence-aligned to the nNOS structure using BLAST then both overlaid CaM-binding helices were oriented within the EM density using the previously published CaM-bound NOS structure as a reference. The iNOS CaM-binding helix [PDB 3GOF] residues were renumbered to match the nNOS residue alignment. This allowed us to map crosslinks not resolved in the nNOS structure to the corresponding iNOS structure. C_α_-C_α_ Euclidean distances were mapped to the resulting model and measured using Chimera.

### Crosslink-guided molecular docking of nNOS and CaM

Crystal structures of dimerized nNOS oxygenase domains [PDB 1ZVL], reductase domain in its open conformation [PDB 1TLL], and full-length CaM [PDB 3HR4] were obtained from the Protein Databank (19, 20, 34). Water, heme, BH_4_, zinc, and Ca^2+^ cofactors were deleted from the structures. All structures were minimized using UCSF Chimera, then uploaded as molecules in the HADDOCK online server (version 2.4-2022.01) (35, 36) with no flexible segments. The position of both chains of the oxygenase domain was fixed at their original position during initial docking to maintain the nNOS_oxy_ dimerization interface. All crosslinks present between CaM and the oxygenase dimer as well as FMN domain and the oxygenase dimer were input as ambiguous restraints to guide docking, oriented to oxygenase domain monomer α or ß as indicated in the Table 5. In accordance with the accepted 27 Å C_α_-C_α_ DSBU linker arm length (11, 12, 13), distances between C_α_ atoms were set with a range of 3 to 27 Å. Interactions between all proteins were set to 1.0. Docking was performed with 1000 rigid body docking iterations, 200 semi-flexible refinements, and 100 final refinements. For simplicity, we present only the structure identified as ‘best’ based on the HADDOCK score in Results.

### Cryo-EM of DBSU-crosslinked or uncrosslinked CaM-bound nNOS homodimer

The nNOS (70 μM) and CaM (60 μM) were incubated in 50 mM Hepes, pH 7.5, containing 100 mM sodium chloride, 1 mM L-arginine, 2 mM CaCl_2_, and 6 mM beta-mercaptoethanol on ice for 20 min before purification by size exclusion chromatography on a WTC-050S5 SEC column (Wyatt Technology Corp) connected to AKTAmicro liquid chromatograph (GE Healthcare). Prior to injecting the sample onto the column, the sample was spin-filtered using a 0.2 μm NanoSep microfiltration centrifugal device at 13,000 rpm for 1.5 min at 4 °C to remove protein aggregates that may have formed. Fractions containing the CaM-bound nNOS homodimer as determined by a 4 to 15% SDSPAGE gel, were used for subsequent cryo-EM experiments. To prepare the DSBU-crosslinked sample, the CaM-bound nNOS was prepared as above, except that 120 μM CaM was used to form the CaM-bound nNOS, and the CaM-bound nNOS was subsequently diluted to 0.5 μM in solution with 50 mM HEPES, pH 7.5, containing 100 mM potassium chloride, 2 mM CaCl_2_, 10 μM BH_4_, and 5% glycerol. The sample was treated with 250 μM DSBU for 20 min at room temperature and quenched as described above. The sample was concentrated to ≥2 mg/ml using Vivaspin concentrators (MWCO 10,000) and subsequently spin-filtered using a 0.2 μm NanoSep microfiltration centrifugal device. The sample was then repurified using a Superose six Inc 3.2/300 column (Cytiva) connected to an AKTA Chromatograph (GE) already equilibrated with 50 mM HEPES, pH 7.5, containing 100 mM potassium chloride, 1 mM L-arginine, 2 mM CaCl_2_, 10 μM BH_4_, and 6 mM beta-mercaptoethanol. Fractions containing DSBU crosslinked CaM-bound nNOS homodimer were determined by a 4 to 15% SDS-PAGE gel and used in subsequent cryo-EM experiments.

The sample of uncrosslinked CaM-bound nNOS homodimer was diluted to 0.18 to 0.21 mg/ml in the absence of reducing agent and then 3.0 μl of sample was applied to a 2/2, 200 mesh, copper quantifoil grid that had been glow-discharged at 15 mA for 120 s. The crosslinked sample was similarly diluted to 0.15 to 0.2 mg/ml and then 3.0 μl of sample was applied to a 1.2/1.3300 mesh copper quantifoil grid, or 3.5 μl was applied to a 1.2/1.3400 mesh gold quantifoil grid. Both grid types were glow-discharged at 15 mA for 120 s before protein application. All samples were vitrified using a Vitrobot (FEI) at 4 °C with 100% humidity, 2 s blot time, 15 s wait time, and a blot force of 0 into liquid ethane. Although we have maintained CaCl_2_ in all buffers used for preparation of the sample, we cannot rule out whether some dissociation of CaM occurred during vitrification for cryo-EM.

Uncrosslinked CaM-bound nNOS was imaged on the Titan Krios 2 TEM operated at 300 keV equipped with a Gatan K2 direct electron detector and Gatan energy filter with a calibrated pixel size of 1.059 Å/pixel. A defocus range of 1.0 to 2.0 μm was used with a total exposure time of 8 s fractionated into 40 subframes for a total dose of ≈ 56 e^−^/Å^2^ at a dose rate of 7.6 e^−^/pixel/s. Movies were motion-corrected for drift using MotionCor2 (37) and were binned by a factor of two for a final pixel size of 2.118 Å/pixel. A total of two datasets were collected using these collection conditions. One additional dataset was collected in which the 8 s exposure time was fractionated into 80 subframes (total dose of ≈ 56 e^−^/Å^2^ at a dose rate of 7.6 e^−^/pixel/s). The crosslinked sample was imaged on the Glacios TEM operated at 200 keV equipped with a Gatan K2 direct electron detector with a calibrated pixel size of 0.912 Å/pixel. A defocus range of 1 to 2 μm was used with a total exposure time of 10 s fractionated into 100 subframes for a total dose of 62 e^−^/Å^2^ at a dose rate of 5.33 e^−^/pixel/s. Movies were motion-corrected for drift using MotionCor2 (37) and were binned by a factor of two for a final pixel size of 1.824 Å/pixel.

All datasets were CTF corrected using CTFFind4 (38). Uncrosslinked CaM-bound nNOS micrographs (≈10,500 micrographs) were visually inspected for sufficient Thon rings and overall image quality using Relion (39) or cisTEM (40). The micrographs (≈14,200 micrographs) from the crosslinked samples were inspected using Scipion (41). Particles for each dataset were then automatically picked using cisTEM and extracted in Relion format for subsequent processing in cryoSPARC2 (42). Approximately 1.86 million particles were extracted from the uncrosslinked samples, and 2.3 million from the crosslinked samples. Each dataset went through three subsequent rounds of 2D classification in cryoSPARC2 to remove contamination, junk particles, and 2D class averages that were not particle-like. The particles selected after 2D classification from each dataset were combined for further 3D classification in cryoSPARC2 (≈875,000 uncrosslinked particles, ≈1.6 million crosslinked particles). Uncrosslinked particles were first sorted into five ab-initio classes. Next, class 03 from these initial five classes was further sorted into two classes. Class 00 from this second round of 3D classification was used for non-uniform refinement to obtain the final oxygenase dimer density. Symmetry was not imposed.

Crosslinked particles were first sorted into five ab-initio classes. Class 01 was sorted into two additional 3D classes after z-axis flip. Non-uniform refinement of class 01 from this second round of 3D classification was performed to obtain the final crosslinked oxygenase dimer density. Symmetry was not imposed. Rosetta Fast Relax (43) was used to fit the nNOS oxygenase dimer crystal structure [PDB 1ZVL] in which two loops between P338:D347 and T466:H470 were remodeled using RosettaES (44) into the final cryo-EM maps generated for the nNOS oxygenase dimer. Ramachandran and bond outliers were corrected using ChimeraX’s ISOLDE (45) and Coot (46). The map and model of the uncrosslinked or DSBU crosslinked CaM-bound nNOS homodimer were validated using Phenix (47).

## Data availability

The mass spectrometry proteomics data along with annotated spectra have been deposited to the ProteomeXchange Consortium *via* the PRIDE partner repository with the dataset identifier PXD044750. Precursor charge data are on Tables S1 and S2. Cryo-EM densities have been deposited at the Electron Microscopy Data Bank under accession codes EMD-40969 (Uncrosslinked nNOS-CaM oxygenase homodimer) and EMD-40970 (DSBU crosslinked nNOS-CaM oxygenase homodimer). Cryo-EM data collection, processing, and validation statistics are given in Table S3. Atomic coordinates have been deposited at the Protein Data Bank under accession codes 8T1J (Uncrosslinked nNOS-CaM oxygenase homodimer) and 8T1K (DSBU crosslinked nNOS-CaM oxygenase homodimer).

## Supporting information

This article contains supporting information.

## Conflict of interest

The authors declare that they have no conflicts of interest with the contents of this article.

## Supporting information

Supporting Table S3

Supporting Figure S1 **Validation of cryo-EM structure of the oxygenase domain derived from uncrosslinked and DSBU-crosslinked nNOS-CaM homodimer.** Cryo-EM map of uncrosslinked (*A*) or DSBU crosslinked (*E*) nNOS-CaM colored by local resolution determined using cryoSPARC2. Gold standard Fourier shell correlation (FSC) curve of uncrosslinked (*B*) or DSBU crosslinked (*F*) nNOS-CaM. 3D angular distribution plot of the particles used in the final reconstruction of uncrosslinked (*C*) or DSBU crosslinked (*G*) nNOS-CaM cryo-EM maps. Map *versus* Model FSC of uncrosslinked (*D*) or DSBU crosslinked (*H*) nNOS-CaM determined using Phenix.
