# Supplementary material for: Mapping interactions of calmodulin and neuronal NO synthase by crosslinking and mass spectrometry
Source: J Biol Chem. 2023 Nov 16;300(1):105464. doi: 10.1016/j.jbc.2023.105464 (PMC10716779; doi:10.1016/j.jbc.2023.105464)
Supplement: Supporting Table S1 [file mmc1.docx]

**Table S1. Crosslinks identified between CaM and nNOS following treatment with DSBU crosslinker.**

| **#** | **CaM Residue** | **nNOS Residue** | **m/z** | **Precursor Charge (z)** | **Score** |
| --- | --- | --- | --- | --- | --- |
| 1 | K22 | K725 | 743.869 | 4 | 128 |
| 2 | T30 | K733 | 700.371 | 4 | 33 |
| 3 | T35 | K613 | 943.779 | 3 | 26 |
| 4 | K95 | Y604 | 883.697 | 4 | 17 |
| 5 | K95 | K771 | 878.193 | 4 | 105 |
| 6 | K95 | S1077 | 910.712 | 4 | 21 |
| 7 | Y100 | K1080 | 911.207 | 4 | 108 |
| 8 | T111 | K302 | 797.916 | 4 | 72 |
| 9 | T111 | K469 | 706.943 | 5 | 77 |
| 10 | K116 | K469 | 883.176 | 4 | 66 |
| 11 | K116 | K725 | 872.68 | 4 | 15 |
| 12 | T118 | K38 | 1089.578 | 3 | 23 |
| 13 | T118 | K725 | 872.68 | 4 | 27 |
