# Supplementary material for: Mapping interactions of calmodulin and neuronal NO synthase by crosslinking and mass spectrometry
Source: J Biol Chem. 2023 Nov 16;300(1):105464. doi: 10.1016/j.jbc.2023.105464 (PMC10716779; doi:10.1016/j.jbc.2023.105464)
Supplement: Supporting Table S2 [file mmc2.docx]

**Table S2. Crosslinks identified within nNOS following DSBU treatment.**

| **#** | **Residue 1** | **Residue 2** | **m/z** | **Precursor Charge (z)** | **Score** |
| --- | --- | --- | --- | --- | --- |
| 1 | *M1 | K38 | 956.023 | 4 | 115 |
| 2 | K24 | K229 | 644.606 | 4 | 105 |
| 3 | K24 | K242 | 528.803 | 4 | 78 |
| 4 | K24 | S368 | 691.381 | 4 | 93 |
| 5 | K33 | S140 | 699.577 | 5 | 101 |
| 6 | K33 | K188 | 657.159 | 5 | 76 |
| 7 | K33 | K225 | 565.316 | 4 | 180 |
| 8 | K33 | S243 | 684.118 | 4 | 75 |
| 9 | K33 | K302 | 501.808 | 4 | 95 |
| 10 | K33 | K452 | 602.339 | 4 | 57 |
| 11 | K33 | K732 | 509.055 | 4 | 90 |
| 12 | S215 | K229 | 872.208 | 4 | 220 |
| 13 | K225 | K302 | 430.249 | 4 | 164 |
| 14 | K225 | K469 | 515.509 | 4 | 65 |
| 15 | K229 | S243 | 585.091 | 5 | 195 |
| 16 | K229 | K406 | 780.144 | 4 | 120 |
| 17 | K229 | K1320 | 607.565 | 4 | 62 |
| 18 | K242 | S367 | 661.586 | 4 | 56 |
| 19 | S243 | K245 | 690.942 | 5 | 67 |
| 20 | K245 | K245 | 731.364 | 4 | 191 |
| 21 | K245 | K302 | 439.443 | 5 | 110 |
| 22 | K245 | K344 | 576.294 | 4 | 102 |
| 23 | K245 | K370 | 622.511 | 5 | 54 |
| 24 | K245 | K620 | 602.817 | 4 | 78 |
| 25 | S280 | K302 | 416.484 | 4 | 108 |
| 26 | T282 | K302 | 554.976 | 3 | 64 |
| 27 | K285 | T289 | 736.87 | 4 | 79 |
| 28 | S287 | K302 | 735.406 | 3 | 72 |
| 29 | T289 | K302 | 441.647 | 5 | 81 |
| 30 | T289 | K370 | 780.393 | 4 | 202 |
| 31 | K290 | K302 | 497.781 | 4 | 185 |
| 32 | S295 | K302 | 483.27 | 3 | 93 |
| 33 | K302 | K344 | 393.986 | 4 | 92 |
| 34 | K302 | T724 | 588.003 | 3 | 81 |
| 35 | K302 | K725 | 441.253 | 4 | 81 |
| 36 | K302 | K733 | 530.002 | 3 | 188 |
| 37 | K302 | K932 | 488.3 | 3 | 142 |
| 38 | K344 | K469 | 479.244 | 4 | 85 |
| 39 | K351 | Y394 | 842.701 | 4 | 152 |
| 40 | K351 | K469 | 467.05 | 5 | 81 |
| 41 | K370 | K406 | 826.67 | 4 | 110 |
| 42 | K406 | K842 | 602.573 | 4 | 67 |
| 43 | K406 | S1410 | 816.675 | 4 | 58 |
| 44 | K452 | K842 | 471.759 | 4 | 195 |
| 45 | K452 | K1302 | 488.77 | 4 | 159 |
| 46 | K469 | K725 | 701.682 | 3 | 214 |
| 47 | K550 | K620 | 538.037 | 4 | 98 |
| 48 | K555 | K613 | 593.802 | 4 | 214 |
| 49 | K612 | K620 | 563.565 | 4 | 163 |
| 50 | K620 | K660 | 738.373 | 5 | 98 |
| 51 | T724 | K732 | 597.667 | 3 | 56 |
| 52 | K725 | K733 | 629.352 | 3 | 160 |
| 53 | K778 | K932 | 602.818 | 4 | 174 |
| 54 | S833 | K856 | 581.284 | 4 | 101 |
| 55 | K842 | S857 | 584.299 | 3 | 67 |
| 56 | K856 | K1320 | 524.775 | 4 | 85 |
| 57 | K932 | S1083 | 840.112 | 3 | 115 |
| 58 | K932 | Y1135 | 666.869 | 4 | 86 |
| 59 | K989 | S1077 | 814.83 | 5 | 81 |
| 60 | K989 | S1083 | 814.629 | 5 | 158 |
| 61 | Y1292 | K1321 | 1293.658 | 3 | 95 |

**Crosslink formed to N-terminal amine of residue M1.*
