# Supplementary material for: Mapping interactions of calmodulin and neuronal NO synthase by crosslinking and mass spectrometry
Source: J Biol Chem. 2023 Nov 16;300(1):105464. doi: 10.1016/j.jbc.2023.105464 (PMC10716779; doi:10.1016/j.jbc.2023.105464)
Supplement: Supporting Table S3 [file mmc3.docx]

**Table S3. Cryo-EM data collection, processing, and validation statistics of the oxygenase dimer derived from uncrosslinked or DSBU crosslinked CaM-bound nNOS oxygenase homodimer.**

|  | Uncrosslinked nNOS-CaM  oxygenase homodimer | DSBU crosslinked nNOS-CaM  oxygenase homodimer |
| --- | --- | --- |
| **Data collection and processing** | | |
| Microscope and camera | Titan Krios, K2 | Glacios, K2 |
| Voltage (kV) | 300 | 200 |
| Data acquisition software | SerialEM | SerialEM |
| Exposure navigation | Image shift | Image shift |
| Electron exposure (e^–^/Å) | 56 | 62 |
| Defocus range (μm) | -2.0 to -1.0 | -2.0 to -1.0 |
| Pixel size (Å) | 1.059 | 0.912 |
| Symmetry imposed | C1 | C1 |
| Initial particle images (no.) | ~ 875,000 | ~ 1,600,000 |
| Final particle images (no.) | 138,304 | 294,924 |
| Map resolution (Å) | 2.73 | 3.14 |
| FSC threshold | 0.143 | 0.143 |
| Map resolution range (Å) | 2.5 – 4.5 | 2.5 – 4.5 |
| **Refinement** | | |
| Model resolution (Å) | 3.0 | 3.2 |
| FSC threshold | 0.5 | 0.5 |
| Map sharpening B factor (Å^2^) | -105.7 | -150.1 |
| **Model composition** | | |
| Nonhydrogen atoms | 6973 | 6973 |
| Protein residues | 839 | 839 |
| Ligands | 7 | 7 |
| **RMSDs** | | |
| Bond lengths (Å) | 0.011 | 0.012 |
| Bond angles (°) | 1.258 | 1.262 |
| **Validation** | | |
| MolProbity score | 0.87 | 0.97 |
| Clashscore | 0.95 | 1.25 |
| Poor rotamers (%) | 0.00 | 0.00 |
| **Ramachandran plot** | | |
| Favored (%) | 97.60 | 97.36 |
| Allowed (%) | 2.40 | 2.64 |
| Disallowed (%) | 0.00 | 0.00 |
