# Supplementary material for: Mapping interactions of calmodulin and neuronal NO synthase by crosslinking and mass spectrometry
Source: J Biol Chem. 2023 Nov 16;300(1):105464. doi: 10.1016/j.jbc.2023.105464 (PMC10716779; doi:10.1016/j.jbc.2023.105464)
Supplement: Supporting Figure S1 — Validation of cryo-EM structure of the oxygenase domain derived from uncrosslinked and DSBU-crosslinked nNOS-CaM homodimer. Cryo-EM map of uncrosslinked (A) or DSBU crosslinked (E) nNOS-CaM colored by local resolution determined using cryoSPARC2. Gold standard Fourier shell correlation (FSC) curve of uncrosslinked (B) or DSBU crosslinked (F) nNOS-CaM. 3D angular distribution plot of the particles used in the final reconstruction of uncrosslinked (C) or DSBU crosslinked (G) nNOS-CaM cryo-EM maps. Map versus Model FSC of uncrosslinked (D) or DSBU crosslinked (H) nNOS-CaM determined using Phenix. [file mmc4.pdf]

Figure S1

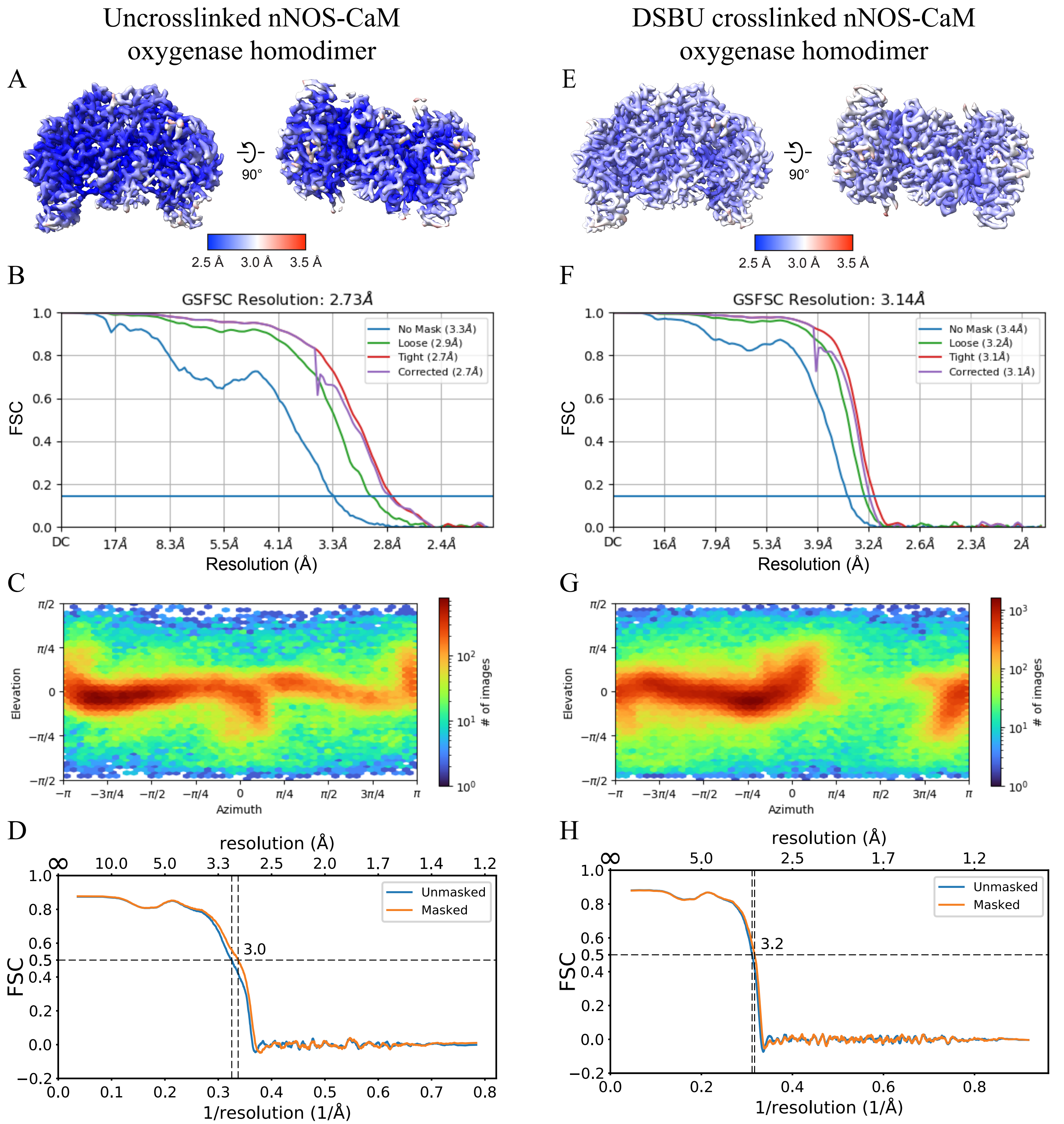

Uncrosslinked nNOS-CaM oxygenase homodimer
DSBU crosslinked nNOS-CaM oxygenase homodimer
A
90°
2.5 Å 3.0 Å 3.5 Å
E
90°
2.5 Å 3.0 Å 3.5 Å
B
GSFSC Resolution: 2.73Å
No Mask (3.3Å)
Loose (2.9Å)
Tight (2.7Å)
Corrected (2.7Å)
FSC
Resolution (Å)
DC 17Å 8.3Å 5.5Å 4.1Å 3.3Å 2.8Å 2.4Å
F
GSFSC Resolution: 3.14Å
No Mask (3.4Å)
Loose (3.2Å)
Tight (3.1Å)
Corrected (3.1Å)
FSC
Resolution (Å)
DC 16Å 7.9Å 5.3Å 3.9Å 3.2Å 2.6Å 2.3Å 2Å
C
Elevation
Azimuth
# of images
G
Elevation
Azimuth
# of images
D
resolution (Å)
Unmasked
Masked
3.0
FSC
1/resolution (1/Å)
H
resolution (Å)
Unmasked
Masked
3.2
FSC
1/resolution (1/Å)
