# Supplementary material for: Mapping interactions of calmodulin and neuronal NO synthase by crosslinking and mass spectrometry
Source: J Biol Chem. 2023 Nov 16;300(1):105464. doi: 10.1016/j.jbc.2023.105464 (PMC10716779; doi:10.1016/j.jbc.2023.105464)

Supporting Information

Figure S2 Representative MS^2^ spectra and sequence assignments for crosslinks listed in Table 1. (A). MS^2^ spectrum for the crosslink between K22 (CaM) and K725 (nNOS)


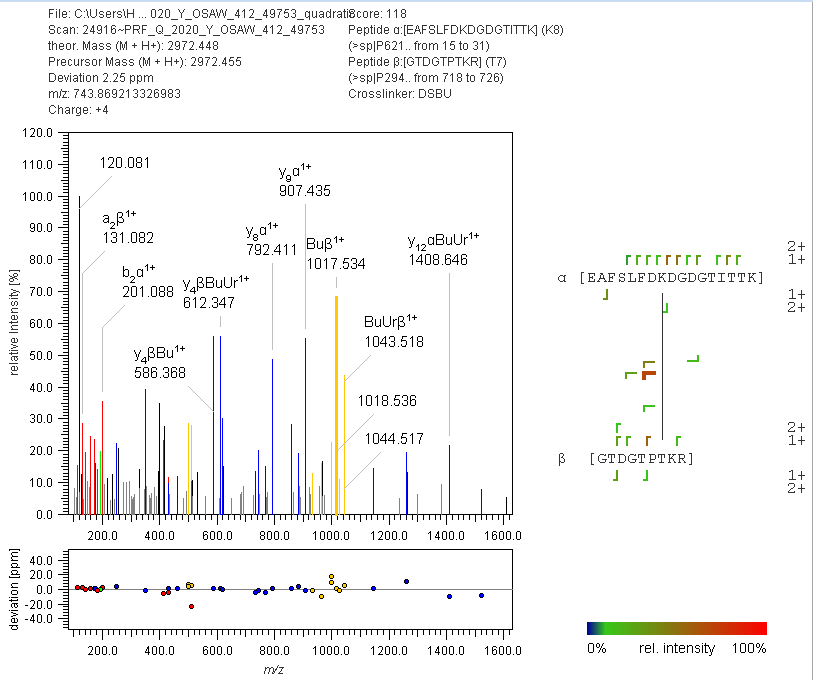


(B). MS^2^ spectrum for the crosslink between K95 (CaM) and K771 (nNOS)


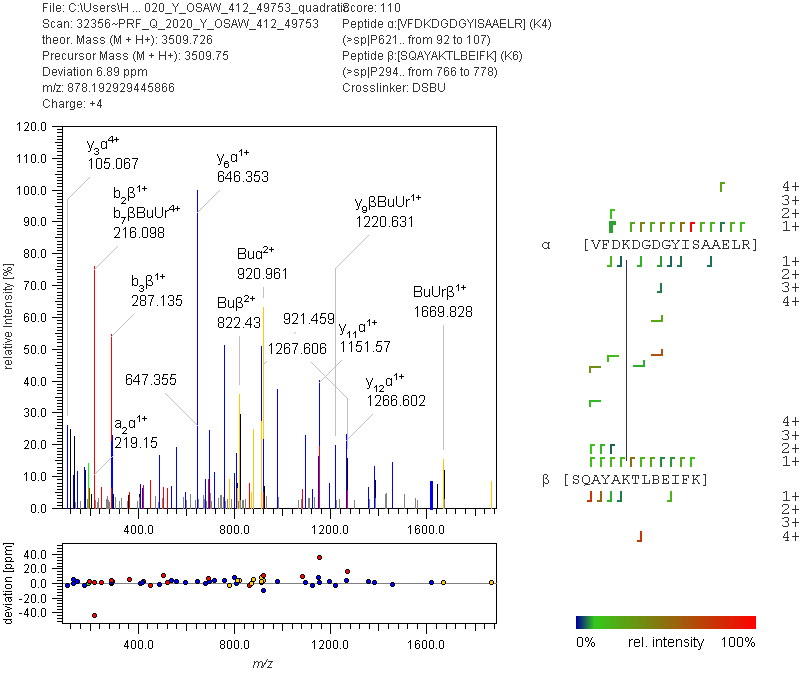

Supplement: Supporting Figure S2 [file mmc5.docx]
